# Crotonylation of key metabolic enzymes regulates carbon catabolite repression in *Streptomyces roseosporus*

**DOI:** 10.1038/s42003-020-0924-2

**Published:** 2020-04-24

**Authors:** Chen-Fan Sun, Wei-Feng Xu, Qing-Wei Zhao, Shuai Luo, Xin-Ai Chen, Yong-Quan Li, Xu-Ming Mao

**Affiliations:** 1 Institute of Pharmaceutical Biotechnology & Research Center for Clinical Pharmacy, The First Affiliated Hospital, School of Medicine, Zhejiang University, 310058 Hangzhou, China; 2 Zhejiang Provincial Key Laboratory for Microbial Biochemistry and Metabolic Engineering, 310058 Hangzhou, China; 3 Zhejiang Provincial Key Laboratory for Drug Evaluation and Clinical Research, 310003 Hangzhou, China

**Keywords:** Microbiology, Molecular biology, Physiology

## Abstract

Due to the plethora natural products made by *Streptomyces*, the regulation of its metabolism are of great interest, whereas there is a lack of detailed understanding of the role of posttranslational modifications (PTM) beyond traditional transcriptional regulation. Herein with *Streptomyces roseosporus* as a model, we showed that crotonylation is widespread on key enzymes for various metabolic pathways, and sufficient crotonylation in primary metabolism and timely elimination in secondary metabolism are required for proper *Streptomyces* metabolism. Particularly, the glucose kinase Glk, a keyplayer of carbon catabolite repression (CCR) regulating bacterial metabolism, is identified reversibly crotonylated by the decrotonylase CobB and the crotonyl-transferase Kct1 to negatively control its activity. Furthermore, crotonylation positively regulates CCR for *Streptomyces* metabolism through modulation of the ratio of glucose uptake/Glk activity and utilization of carbon sources. Thus, our results revealed a regulatory mechanism that crotonylation globally regulates *Streptomyces* metabolism at least through positive modulation of CCR.

## Abstract (web summary)

Chen-Fan Sun et al. show that key enzymes in several metabolic pathways are crotonylated in *Streptomyces roseosporus*. This study suggests that crotonylation increases carbon catabolite repression by increasing glucose uptake while reducing the activity of glucose kinase.

## Introduction

*Streptomyces* are profoundly famed as bacterial producers of natural products with diverse chemical structures and bioactivities^1^. The short-chain acyl-CoA species, such as acetyl-CoA, malonyl-CoA, and succinyl-CoA, are catabolites from primary metabolism. Meanwhile, they are common precursors and building blocks for biosynthesis of macromolecules in primary metabolism and natural products in secondary metabolism^2–4^. It has been well recognized that precise control of primary/secondary metabolism development of *Streptomyces* and their switch are critical for the proper production of these invaluable natural products^5^.

Traditionally, regulation of *Streptomyces* metabolism has been extensively studied at the transcriptional levels^6,7^. But recently, protein acetylation from acetyl-CoA, one of the posttranslational modifications (PTMs), has been shown to be involved in the regulation of production of both primary and secondary metabolites in *Streptomyces*. Acetylation was initially reported in two conserved *Streptomyces* enzymes acetyl-CoA synthetase^8^ and acetoacetyl-CoA synthetase^9^. Following acetyl-proteome assays showed that acetylation is widely distributed in *Streptomyces*, since 667 and 134 proteins were identified acetylated in *S. roseosporus* and *S. griseus*, respectively. Acetylated proteins are mainly involved in metabolism, followed by protein biogenesis and turn-over. Meanwhile, acetylation also occurs on some biosynthetic enzymes for natural products, including the dTDP-4-dehydrorhamnose 3,5-epimerase StrM for streptomycin biosynthesis and the adenylation domain of a non-ribosomal peptide synthase^10,11^. Interestingly, acetylation of enzymes unexceptionally abolishes their catalytic activities^8,9,11^. Though regulation of PTMs on the activity of some enzymes has been reported as shown above, how PTMs regulate the *Streptomyces* metabolism in a broader scope has not yet been addressed.

Carbon catabolite repression (CCR) is a conserved mechanism allowing the bacteria, as well as *Streptomyces*, to generally utilize preferred carbon source (such as glucose in laboratory cultivations) over others and attracting the attention as a checkpoint for differentiation of *Streptomyces*^12^. Glucose induction often triggers CCR and leads to delayed morphological development and repressed secondary metabolite production^13–15^. In *Streptomyces*, glucose kinase (Glk) has been shown to be involved in CCR regulation^16,17^, possibly through its kinase activity to phosphorylate glucose and strengthen glycolysis to produce metabolic intermediates for CCR regulation and through its regulatory activity by interacting with downstream transcriptional regulators^13,18,19^. It has been proposed that the cytosolic fraction of Glk has a certain kind of PTM for its interaction with regulators, while Glk can also be recruited to the cell membrane to interact with the permease GlcP for glucose metabolism^20^. Meanwhile, the ratio of glucose uptake/Glk kinase activity has been suggested as a determinant of sensitivity to CCR^18^. However, there is no report about which PTM of Glk for CCR regulation on *Streptomyces* metabolism.

To our knowledge, crotonylation is a newly identified PTM on eukaryotic histones^21^. Modification of histones with this unique planar, four-carbon structure, and neutral charge reduces histone–DNA interaction^22^. Thus histone crotonylation is predominantly found on transcriptionally active regions and enhancers during mouse spermatogenesis^21^, renewal of embryonic stem cells^23^, and acute kidney injury^24^. In addition to its epigenetic modification on histones, crotonylation also occurs in a broad range of non-histone proteins^25–28^ and participates in diverse metabolic pathways such as acetylation^29^. Crotonylation happens through reversible modifications by enzymes, including crotonyltransferases (writers) and decrotonylases (erasers). Studies have shown that crotonylation has overlapped acyl-transferases and de-acylases with acetylation and other types of acylations^25,30^ and also overlapped modification sites on histones^29,31^. These findings suggest that crotonylation is a global PTM with a complex interplay with other acylations. Nevertheless, crotonylation in bacteria, as well as how it regulates the bacterial metabolism, has not been reported.

Here we showed that crotonylation is universal in *Streptomyces* metabolic pathways and regulates the metabolism at least through positively modulating CCR, by reversible modifications on Glk. Our work provides comprehensive insights by far, exploring the mechanisms of PTM in regulating *Streptomyces* metabolism, and potentially paves the way for PTM engineering in *Streptomyces* for optimal production of secondary metabolites.

## Results

### Protein crotonylation is widespread in *Streptomyces*

*Streptomyces* proteins have been found extensively acetylated, and this PTM plays essential roles in enzyme activity modulation for primary/secondary metabolite biosynthesis^8–11^. Though acetyl-CoA is believed the most abundant acyl-CoA species^32^, other acyl-CoAs might have more subtle and complex roles in the regulation of *Streptomyces* metabolism.

**Fig. 1:**
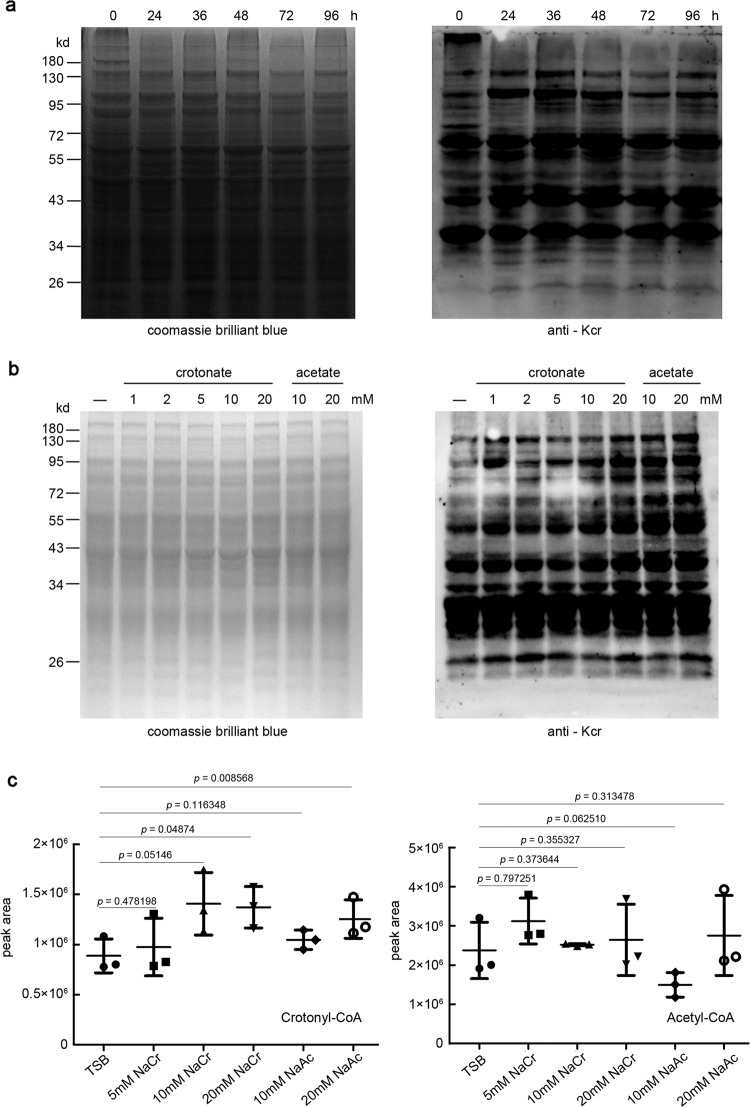
Crotonylation in *S. roseosporus*. **a** Immunoblot of *S. roseosporus* lysate. Twenty μg of total protein were loaded, and the crotonylated proteins were detected with anti-Kcr monoclonal antibody (1:2000) while Coomassie blue staining was used for the loading control. **b** Immunoblot for crotonylation with anti-Kcr monoclonal antibody (1:2000) in wild-type strain cultured in TSB with additional concentration of sodium crotonate (pH 7.4) or sodium acetate (pH 7.4) as indicated for 24 h. Twenty μg of total protein were loaded, and Coomassie blue staining was used for the loading control. **c** LC-MS analysis of cellular crotonyl-CoA and acetyl-CoA levels from cells cultured as in **b**. The data represent mean peak area ±SD of three independent experiments. *P* value was calculated with Student’s *t* test (ns means *P* > 0.05).

When exploring the PTM profile of *Streptomyces* proteome throughout the fermentation, we found strong signals from immunoblots with an anti-lysine-crotonyl group (α-Kcr) antibody, as represented by an antibiotic daptomycin producer *S. roseosporus* (Fig. 1a), as well as laboratory model species like *S. coelicolor* M145^33^ and *S. lividans* TK24^34^, and the industrial producers *S. albus* J1074^35^ and *S. tsukubaensis* L19^36^ (Supplementary Fig. 1), suggesting that crotonylation occurs widely in *Streptomyces*. In *S. roseosporus*, the signals from cultures at 36 and 48 h were much stronger, when cells were entering secondary metabolism, than that at 24 h or after 48 h, implying that crotonylation increases during primary metabolism development but decreases during secondary metabolism development. The crotonylation dynamics suggested that this PTM might have regulatory roles on *Streptomyces* primary/secondary metabolism development.

Using this antibody, we detected obviously increased crotonylation levels in cells treated with gradient concentrations of sodium crotonate (NaCr). Meanwhile, sodium acetate (NaAc) treatment also resulted in apparently increased crotonylation (Fig. 1b). However, crotonate or acetate treatments had little impact on acetylation or succinylation (Supplementary Fig. 2). These data suggested that crotonylation is more subtle to environmental signals. Furthermore, we examined the dynamics of crotonyl-CoA and acetyl-CoA levels in response to NaCr or NaAc by liquid chromatography–mass spectrometry (LC-MS; Fig. 1c). Increase of crotonyl-CoA concentration was observed only under 20 mM NaCr or NaAc, whereas no obvious increase of the intra-cellular acetyl-CoA was observed, suggesting that acetyl-CoA is resistant to environmental NaAc fluctuation, while intracellular crotonyl-CoA is mainly derived from acetyl-CoA^37^.

### Crotonylation occurs on various metabolic pathways and regulators

**Fig. 2:**
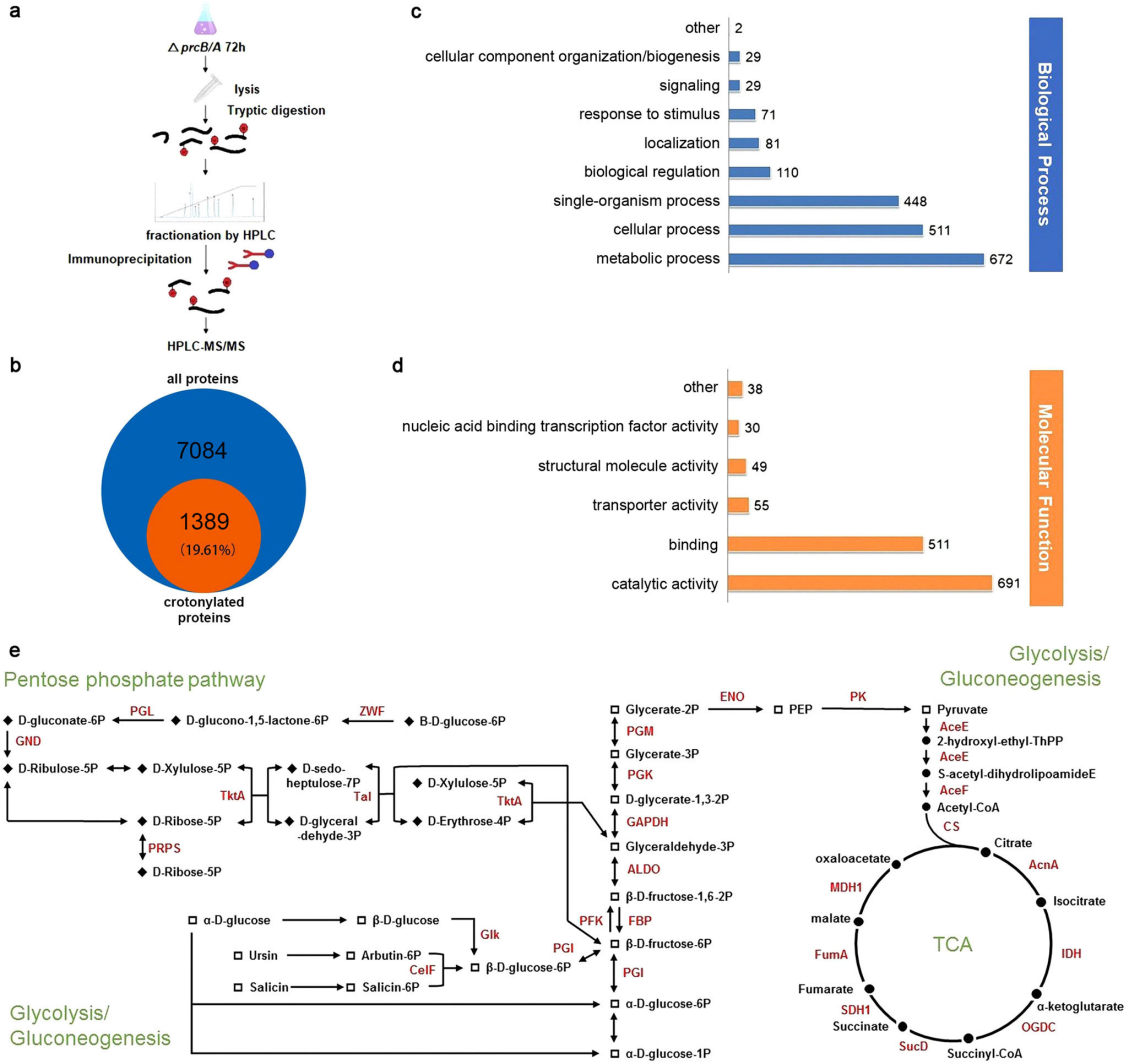
Crotonyl-proteomic analysis of *S. roseosporus* proteins. **a** Procedure diagram for crotonyl-proteomics in *S. roseosporus*. **b** Statistics of crotonylated proteins from *S. roseosporus*. **c**, **d** Gene ontology functional classification of the identified crotonylation proteins based on biological processes (**c**) and molecular functions (**d**). **e** Carbon metabolism pathways in *Streptomyces*. Crotonylated enzymes are highlighted in red.

Next crotonyl-proteomics were performed in *S. roseosporus* to identify the substrates for crotonylation. Since proteasome-mediated degradation is one of the major pathways for protein turn-over^38^, a proteasome-deficient *ΔprcB/A* mutant was constructed (Supplementary Fig. 3) to potentially enrich more proteins with PTMs by immunoprecipitation with the anti-Kcr monoclonal antibody, and crotonylated proteins along with the modified residues were identified by high-performance LC-MS/MS (Fig. 2a). All the distribution of mass error for precursor ions was <0.03 Da, indicating acceptable mass accuracy of the MS data (Supplementary Fig. 4, Supplementary Data 3). Strikingly, 1389 proteins, along with 3944 lysine residues, were identified with crotonylation, accounting for 19.61% of the putative proteome of *S. roseosporus* (Fig. 2b) (Supplementary Data 2 and 3). It was a contrast to the previous acetyl-proteomic survey in the same species *S. roseosporus* NRRL 11379, where 667 acetylated proteins and 1143 modification sites were identified^10^.

Gene ontology (GO) analysis (Supplementary Fig. 5, Supplementary Data 4) showed that 672 crotonylated proteins (34%) are involved in the metabolic processes (Fig. 2c), while 691 crotonylated proteins (50%) are enzymes with catalytic activities (Fig. 2d), suggesting that crotonylation primarily regulates enzymatic activities in *S. roseosporus* metabolism. Consistent with these observations, Kyoto Encyclopedia of Genes and Genomes (KEGG) analysis (Supplementary Data 5) showed that most enzymes in carbon metabolism are crotonylated (Supplementary Fig. 6). Particularly, all enzymes in glucose catabolism, including glycolysis, pyruvate dehydrogenation, and tricarboxylic acid cycle (TCA) (Fig. 2e, Supplementary Figs. 7 and 8), and most enzymes in oxidative phosphorylation are highly crotonylated (Supplementary Data 5). These data strongly suggested that crotonylation globally regulates glucose utilization, respiratory chain, and potentially production of primary metabolites, such as acetyl-CoA, NADH, NADPH, and ATP. Other critical metabolic pathways in primary metabolism strongly regulated by crotonylation are DNA replication and repair, RNA biogenesis and degradation, protein biogenesis, and turn-over. It has been found that DNA polymerase, topoisomerase, gyrases and UvrABC, RNA polymerase and ribonucleases, most ribosomal proteins, almost all aminoacyl-tRNA synthetases, three chaperones, and protein degradation system (protease Clp, Lon, proteasome components) are heavily modified (Supplementary Figs. 9 and 10, Supplementary Data 3). Moreover, some acyl-CoA synthetases, including acetyl-CoA synthetase AcsA, are also crotonylated, but interestingly, no de-acylases are found modified with crotonylation (Supplementary Data 3), suggesting that crotonylation might modulate the biogenesis and intracellular concentrations of acyl-CoAs.

Meanwhile, regulators for secondary metabolism development were also found crotonylated, such as the putative γ-butyrolactone receptor ArpA^39,40^, the intracellular ppGpp synthase RelA^41^, and the nutrient-responsive regulators GlnR^42^, PhoP^43^, and AtrA^40^ (Supplementary Data 3). More interestingly, crotonylation was also found on some biosynthetic enzymes for natural products, such as DptE, DptA, and DptD, the core enzymes in biosynthesis of daptomycin (Supplementary Data 3), a representative antibiotic produced by *S. roseosporus*^40,44^. All these data suggested that crotonylation globally occurs on key metabolic enzymes, biosynthetic enzymes, and critical regulators and thus might regulate metabolism of *Streptomyces*.

Among all PTMs identified to date, acetylation has been extensively mapped in diverse organisms. We compared the crotonylation substrates identified in our study to previously determined acetyl-proteome in *S. roseosporus*^10^. We found that 37% of crotonylated proteins are acetylated as well at their Lys residues (Supplementary Fig. 11a). Meanwhile, these two acylations possesses priority of various modification-occurring motifs (Supplementary Fig. 11b). Further KEGG pathway enrichment (Supplementary Fig. 11c) and protein domain enrichment (Supplementary Fig. 11d) of these two modifications suggested that the most enriched categories for crotonylated proteins were ribosome (*p* = 2.40E−09) and carbon metabolism, such as citrate cycle (*p* = 2.14E−07), glycolysis/gluconeogenesis (*p* = 4.22E−05), and pyruvate metabolism (*p* = 3.59E−06) (Supplementary Fig. 11c), whereas they were aminoacyl-tRNA biosynthesis (*p* = 2.33E−06) and ribosome (*p* = 3.19E−05) for acetylation.

### Crotonylation regulates *Streptomyces* metabolism

**Fig. 3:**
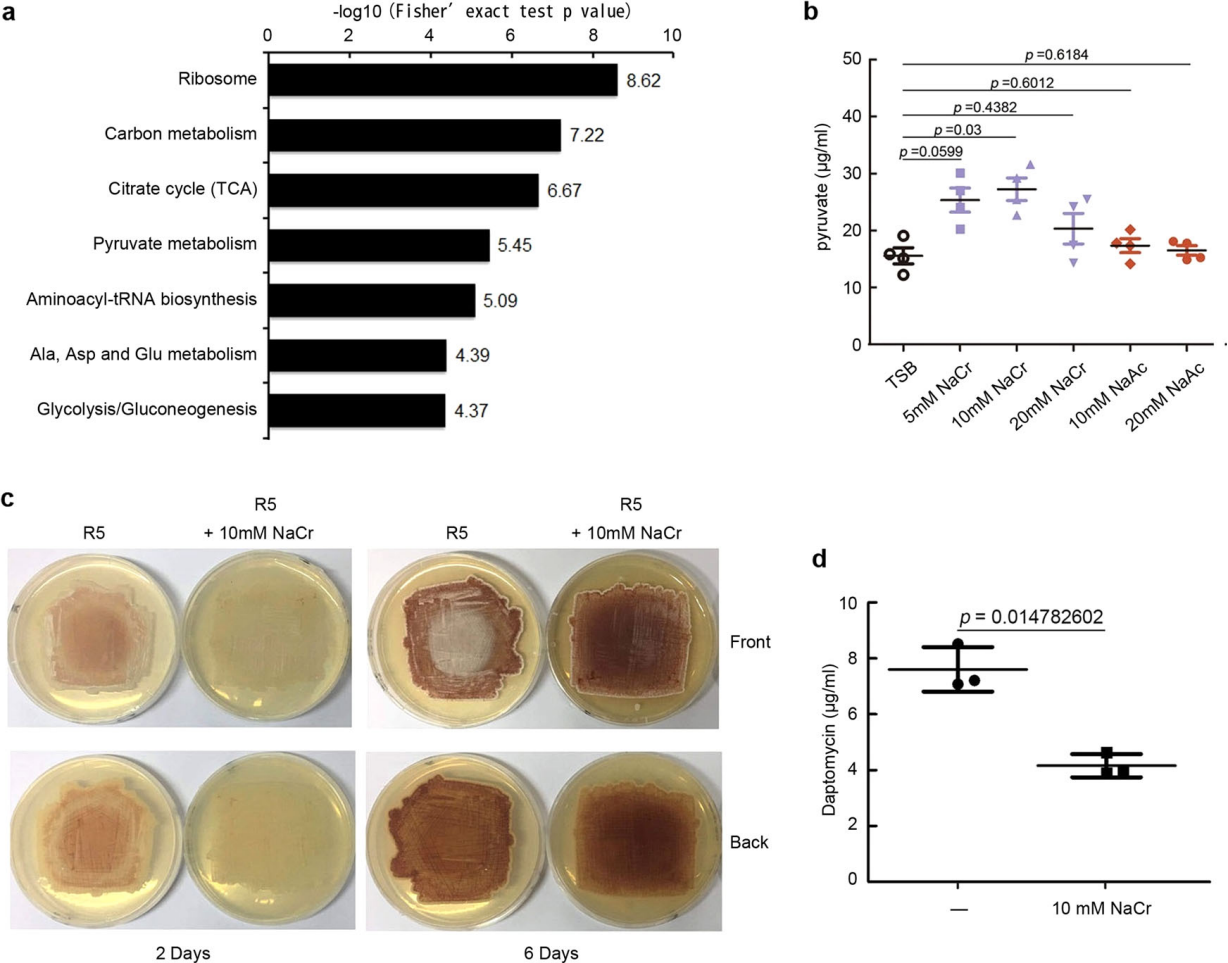
Crotonylation regulates *S. roseosporus* metabolism. **a** KEGG pathway-enrichment analysis for lysine-crotonylated proteins. **b** Pyruvate production assays with addition of NaCr or NaAc. *S. roseosporus* wild type was cultured in TSB supplemented with NaCr (pH 7.4) or NaAc (pH 7.4) with the indicated concentrations for 24 h, and the intracellular pyruvate concentration was determined. *P* value was calculated with Student’s *t* test (*n* = 4). **c** Morphological phenotype of *S. roseosporus* wild type on R5 with or without 10 mM NaCr (pH 7.4) for 2 or 6 days. **d** Daptomycin production in wild type supplemented with 10 mM NaCr. The daptomycin yield was measured after culturing 120 h. Experiments were performed in triplicate. *P* value was calculated with Student’s *t* test (*n* = 3).

Next, we further investigated the regulatory roles of crotonylation for *Streptomyces* metabolism. In the KEGG metabolic pathway analysis (Fig. 3a), the top enriched categories for lysine-crotonylated substrates were ribosome and carbon metabolism, such as TCA cycle, while the top categories of acetylation were aminoacyl-tRNA biosynthesis, ribosome in our strain as well as *S. erythraea*^45^, and ribosome and pyruvate metabolism for succinylation in *Escherichia coli*^46^, implying a possible link between crotonylation and carbon metabolism. Consistently, increased production of pyruvate was observed at the supplement of 10 mM NaCr, but not at 5 or 20 mM NaCr or with addition of NaAc (Fig. 3b). Considering an incremental level of crotonylation during primary metabolism (Fig. 1a), these results suggested that proper enhancement of crotonylation might benefit *Streptomyces* during primary metabolism, but excessive modification would be backfired. Meanwhile, we observed delayed aerial mycelium development (Fig. 3c) and postponed secondary metabolism development for red pigment production (Fig. 3c) and daptomycin production (Fig. 3d) if cells were supplemented with 10 mM NaCr. These observations experimentally suggested that crotonylation is involved in the regulation of both primary and secondary metabolism of *Streptomyces*.

### Identification of modification enzymes for *Streptomyces* crotonylation

**Fig. 4:**
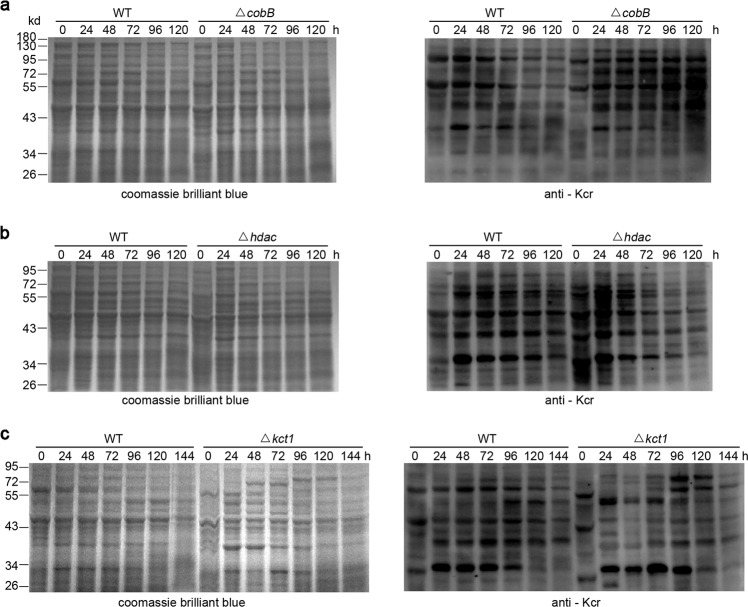
Identification of crotonylation enzymes. Immuno-blot assays of crotonylation with anti-Kcr monoclonal antibody (1:2000) from *S. roseosporus* strain wild type (WT), two decrotonylase mutants *ΔcobB* (**a**) and *Δhdac* (**b**), and a crotonyl-transferase mutant *Δkct1* (**c**) with anti-Kcr monoclonal antibody in right, together with Coomassie blue staining in left for the loading control.

Next, we tried to identify enzymes responsible for this PTM. Protein crotonylation is reversibly catalyzed by crotonyl-transferase (writers) and decrotonylase (erasers)^25,47^, and the canonical acetyltransferase p300 in mammalian cells also acts as a crotonyl-transferase^21,32,48^, suggesting that some enzymes are pluripotent in acyl-modifications. In *Streptomyces*, two deacetyltransferases, the NAD^+^-dependent deacetylase CobB and Zn^2+^-dependent deacetylase histone deacetylase (HDAC), and a putative GCN5-family acetyl-transferase Kct1, have been well characterized^49^ and are highly conserved (Supplementary Figs. 12–14). We found that removal of these homologs in *S. roseosporus* showed remarkable alterations of crotonylation (Supplementary Figs. 15–17). The *ΔcobB* mutant displayed constitutively higher crotonylation during fermentation, particularly after 48 h, than the wild type, whose modification obviously dropped at that time (Fig. 4a and Supplementary Fig. 18a). The *Δhdac* mutant showed more crotonylation in the early developmental phases but lower modification after 48 h compared to the wild type (Fig. 4b and Supplementary Fig. 18b). These data genetically confirmed that both CobB and HDAC work as decrotonylases in *S. roseosporus*, but they primarily function in different developmental stages. HDAC is responsible for the maintenance of low crotonylation in the primary metabolism, while CobB mainly works in the later phases of secondary metabolism. Moreover, deletion of *kct1* caused decreased crotonylation as expected, especially in the early stages before 72 h (Fig. 4c and Supplementary Fig. 18c), but similar protein modification was observed after 72 h between wild type and the *Δkct1* mutant, genetically confirming that Kct1 is a crotonyl-transferase.

Consistent with the altered crotonylation profiles, both *ΔcobB* and *Δhdac* mutants showed more biomass than wild type, while *Δkct1* mutant had retarded growth before 72 h but restored afterwards Supplementary Fig. 19a), further suggesting that sufficient crotonylation in the early phases benefits cell vegetative growth, which was in agreement with the gradually increased crotonylation observed in wild type. Consistently, over-produced pyruvate and ATP in both *ΔcobB* and *Δhdac* mutants but a decreased ATP concentration in the *Δkct1* mutant was observed (Supplementary Fig. 19b). But interestingly, statistically increased production of crotonyl-CoA and decreased production of acetyl-CoA were observed in *Δhdac* and *ΔcobB* mutants, respectively (Supplementary Fig. 19c). Furthermore, both decrotonylase mutants began to produce the secondary metabolite daptomycin at 48 h, earlier than wild type. But it was noteworthy that, though similar daptomycin titers were observed in *ΔcobB* mutant and wild type at 72 and 96 h, the metabolite production in *ΔcobB* mutant dropped much faster afterward, while the *Δhdac* mutant showed constitutively higher productivity (Supplementary Fig. 19d), implying that excessive crotonylation in the early phases might benefit production of secondary metabolites, but crotonylation should be partially erased for proper metabolic development in the later phases. This hypothesis could also explain the observation in wild type, where crotonylation gradually increased before 36 h but slowly dropped after 48 h. Consistently, the *Δkct1* mutant with weakened crotonylation only initiated daptomycin production at 96 h and constantly showed a much lower yield of daptomycin throughout the fermentation (Supplementary Fig. 18d). These data supported that delayed crotonylation postpones cell development and reduces secondary metabolite production.

Some writers and erasers in bacteria are versatile in modifications of proteins with various acylations. For examples, CobB has been shown as a de-acetylase in *E. coli* and as a de-hydroxybutyrylase in *Proteus mirabilis*^50^. Acetylation and succinylation are two representative PTMs in bacteria. However, we found that, in *S. roseosporus*, *ΔcobB* mutant had no obvious change in acetylation profile but showed much higher succinylation, while *Δhdac* mutant had stronger signals both in acetylation and succinylation (Supplementary Fig. 20a). Moreover, no distinct changes were observed in *Δkct1* mutant (Supplementary Fig. 20b). All these data suggested that both erasers also function in other types of acylation of *Streptomyces*, while the writer Kct1 not (Supplementary Fig. 20c). Though we could not exclude the possible involvement of acetylation or succinylation for metabolic changes of *ΔcobB* and *Δhdac* mutants, our observations from *Δkct1* mutant and phenotypes with NaCr addition still supported regulation of crotonylation on *Streptomyces* metabolism.

### Glk is reversibly crotonylated by CobB and Kct1

**Fig. 5:**
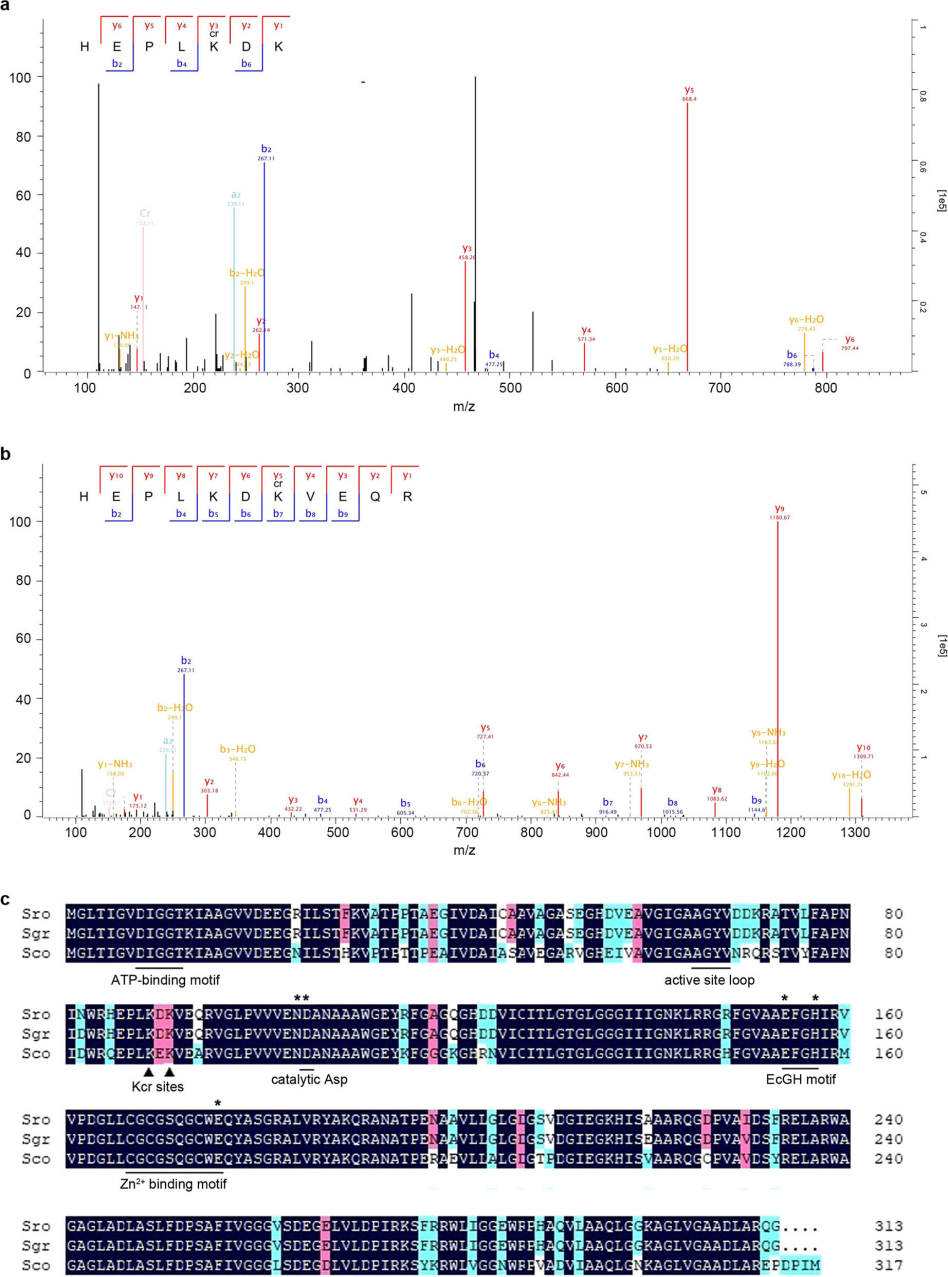
Glk is crotonylated at two conserved lysine (K) residues. **a** MS/MS analysis of crotonylation on Glk K89. Crotonylation was calculated based on the molecular weight difference values between y3 and y2 (458.26 − 262.14 = 196.12), and b6 and b4 (788.39 − 477.25 = 311.14). **b** MS/MS analysis of crotonylation on Glk K91, which was calculated based on the molecular weight difference values between y5 and y4 (727.41 − 531.29 = 196.12), and b7 and b6 (916.49 − 720.37 = 196.12). **c** Glk protein alignment from *S. roseosporus* (Sro), *S. griseus* (Sgr), and *S. coelicolor* (Sco). Two crotonylated residues (Kcr sites) along with conserved motifs are shown. Residues involved in the glucose-binding mechanism were labeled with asterisks.

To better understand the molecular regulatory mechanism of crotonylation and further confirm that above phenotypic alterations on *Streptomyces* metabolism authentically result from crotonylation, we looked for critical substrates that are crotonylated but not acetylated and mediate the regulation of crotonylation on *Streptomyces* metabolism. Here we focused on Glk, due to the global modification of glucose catabolism pathway (Fig. 2e) and given that Glk is the first enzyme to activate glucose and a key regulator of CCR in *Streptomyces*^16,20^. Moreover, Glk was identified crotonylated at two conserved residues K89 and K91 based on MS/MS data (Fig. 5), adjacent to a catalytic D105^51^. But interestingly, no acetylation was found on Glk, which thus excludes the influence of acetylation on Glk in our study.

**Fig. 6:**
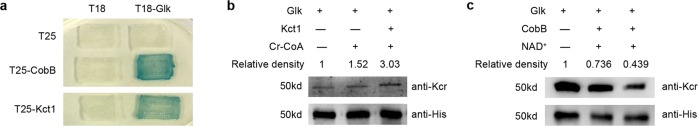
Glk is reversibly crotonylated by CobB and Kct1. **a** Bacterial two-hybrid analysis of the interaction between Glk and CobB or Kct1. Glk was constructed in pT18, while CobB or Kct1 in pT25. *E. coli* strain BTH101 containing the hybrids were cultured on LB for 48 h and photographed. **b**, **c** In vitro assays for Glk crotonylation by Kct1 (**b**) and decrotonylation by CobB (**c**). Reactions were analyzed by western blot with the indicated antibodies (1:2000). The relative intensity of each sample is also shown. In **b**, the reaction time is 1 h, while in **c**, the reaction time for decrotonylation is: lane 1, 1.5 h; lane 2, 1 h; lane 3, 1.5 h.

To identify the enzymes that modify Glk, we constructed a bacterial two-hybrid library comprising 54 putative acyl-transferases and 2 de-acylases as the preys (Supplementary Data 6), while Glk was the bait in this system. Interestingly, CobB and Kct1 were screened out from this library to specifically interact with Glk, since the blue lawn was observed only when Glk co-existed with CobB or Kct1 in one bacterial cell to activate the expression of β-galactosidase in the bacterial two-hybrid system (Fig. 6a). Glk, CobB, and Kct1 were expressed and purified from *E. coli*. Based on the immunoblots with α-Kcr antibody, we found that the purified Glk protein has already been modified with crotonylation (Fig. 6b and Supplementary Fig. 21a, Fig. 6c and Supplementary Fig. 21b). In our in vitro assays, Glk can be further crotonylated with crotonyl-CoA in an enzyme-independent pathway for PTM. However, strong crotonylation was observed only when the acyl-transferase Kct1 was further added, confirming that Glk can be crotonylated by Kct1 (Fig. 6b and Supplementary Fig. 21a). Moreover, when Glk was incubated with CobB and the co-factor NAD^+^, decreased crotonylation was observed with about 27% loss for 1 h and over half for 1.5 h (Fig. 6c and Supplementary Fig. 21b), confirming that CobB is an NAD^+^-dependent decrotonylase to erase crotonylation on Glk.

### Glk activity is regulated by crotonylation

**Fig. 7:**
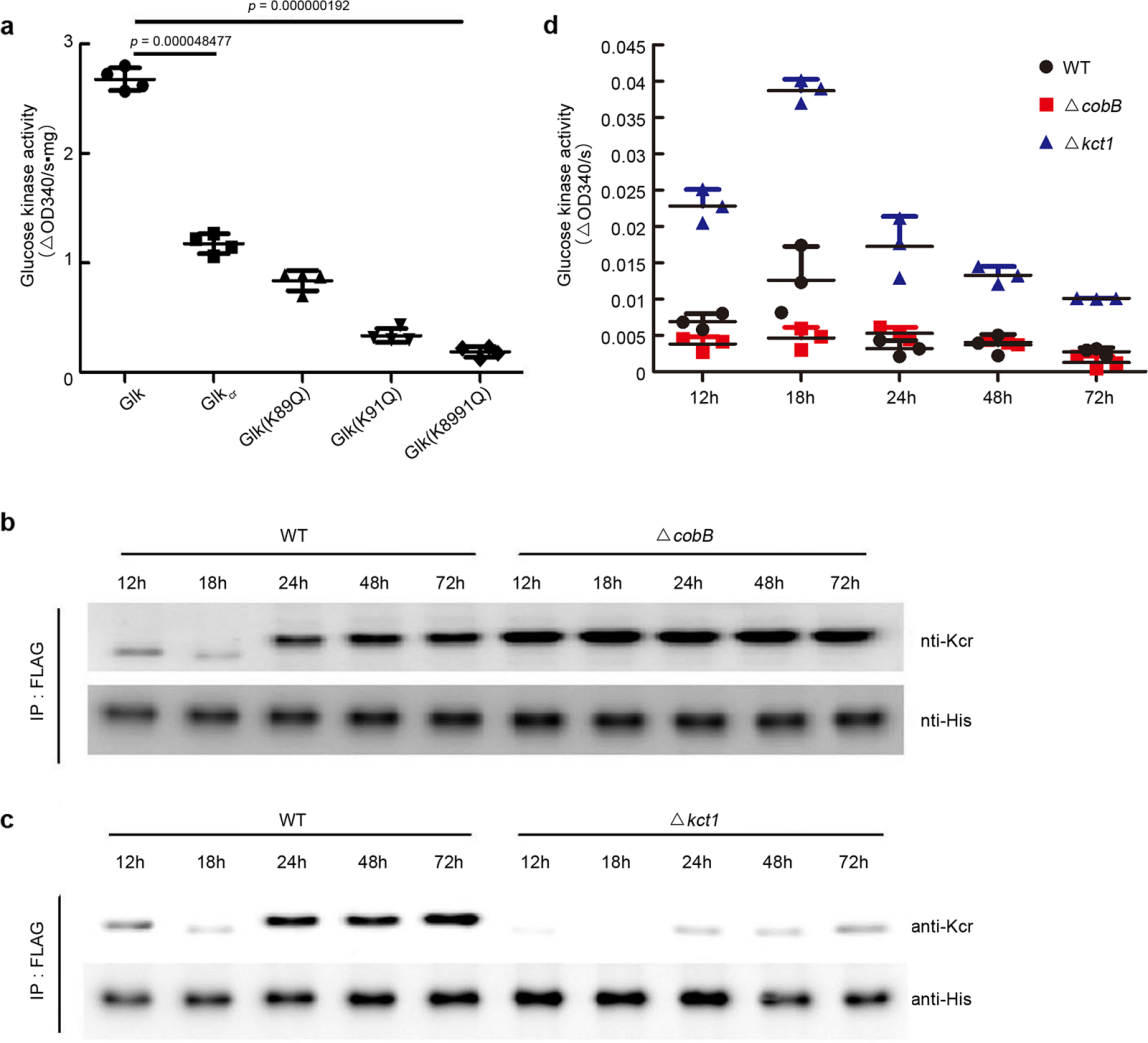
Crotonylation regulates Glk activity. **a** In vitro assays of crotonylation on the Glk catalytic activity. Glk_cr_ was prepared from the in vitro reaction with Glk and Kct1 in Fig. 3b. Glk mutant isoforms were purified from *E. coli*. Statistical significance was determined by two-tailed unpaired Student’s *t* test (*n* = 4). **b**, **c** Western blot analysis of crotonylation level of Glk in vivo. *S. roseosporus* wild type and *ΔcobB* and *Δkct1* mutants expressing ermEp*-3flag-glk were cultured in the YEME medium, and mycelia were taken at the time points indicated. 3FLAG-Glk was immune-precipitated from the lysate, and western blots were performed with anti-FLAG M2 or anti-Kcr rabbit antibody (1:2000). **d** Glucose kinase activity assays of the lysate from wild type and *ΔcobB* and *Δkct1* mutants. Bacterial strains were cultured in the YEME medium, and the kinase activity was measured every 12 h. Experiments were performed in triplicate.

Next, the effects of crotonylation on Glk kinase activity were evaluated. As shown from in vitro assays in Fig. 7a, we found that Glk with higher crotonylation (modified by Kct1 as in Fig. 6b) had lower activity than the native one purified from *E. coli*, which could recover after decrotonylation by CobB (Supplementary Fig. 22a), suggesting that crotonylation reduces Glk activity. Consistent with this observation, crotonylation mimic of these sites by K to Q single mutation also reduced the activity, and the double mutant had a much lower level of kinase activity (Fig. 7a). Consistent with these observations, when *Δglk* mutant was complemented with wild-type Glk protein, the constitutively modified Glk (K8991Q) and the unmodified form (K8998R), we found that K–Q mutation led to lower levels of Glk activity, while K–R mutation accounted for the higher kinase activity based on the whole-cell lysate assays (Supplementary Fig. 22b, c).

Further in vivo investigation of crotonylation in wild type showed that crotonylation of Glk increased during cell metabolism development. In primary metabolism (12 and 18 h), the modification was low but sharply increased at 24 h and moderately increased at 48 and 72 h. However, the lowest crotonylation was observed at 18 h (Fig. 7b and Supplementary Fig. 23a and Fig. 7c and Supplementary Fig. 23b). The dynamics of Glk modification in wild type also correlated to the kinase activity during cell development. Higher activity was observed at primary metabolism, and cells at 18 h had the highest Glk kinase activity (Fig. 7d). These phenomena were well coincident with the previous report in *S. coelicolor* that Glk activity had a peak at 18 h but dropped and was constant afterward^20^. Interestingly, constitutive hyper-crotonylation of Glk was found in the *ΔcobB* mutant throughout the cell development. Especially at 12 and 18 h, this mutant had even much higher modification than wild type from the late development phases (Fig. 7b). This excessive modification of Glk in the *ΔcobB* mutant accounted for the lower activity (Fig. 7d). Moreover, ultra-low modification of Glk was found in the *Δkct1* mutant in all developmental phases, and it was almost undetectable at 18 h (Fig. 7c). As expected, we observed much higher Glk activity in this mutant, particularly at 18 h (Fig. 7d). All these data suggested that crotonylation negatively regulates Glk kinase activity, and the in vivo precise proportion between crotonylation and Glk activity dynamics also suggested that crotonylation might be one of the major PTM mechanisms for Glk activity regulation.

### Positive modulation of CCR by crotonylation

CCR is a conserved regulatory mechanism to utilize one carbon source (such as glucose) to suppress the metabolism of other carbon sources and *Streptomyces* metabolism, and mainly depends on Glk^5,13,14^. Glk kinase activity is required for glucose activation and subsequent glycolysis. It has been shown that there was a global loss of CCR in the *glk*-deletion strain^4^, but both Glk-dependent and independent pathways have been proposed for CCR^16–19^. However, one possible mechanism of Glk on CCR has been proposed that Glk might interact with other transcriptional factors after it is posttranslationally modified^20^. So we further investigated whether crotonylation influences CCR in *Streptomyces*.

**Fig. 8:**
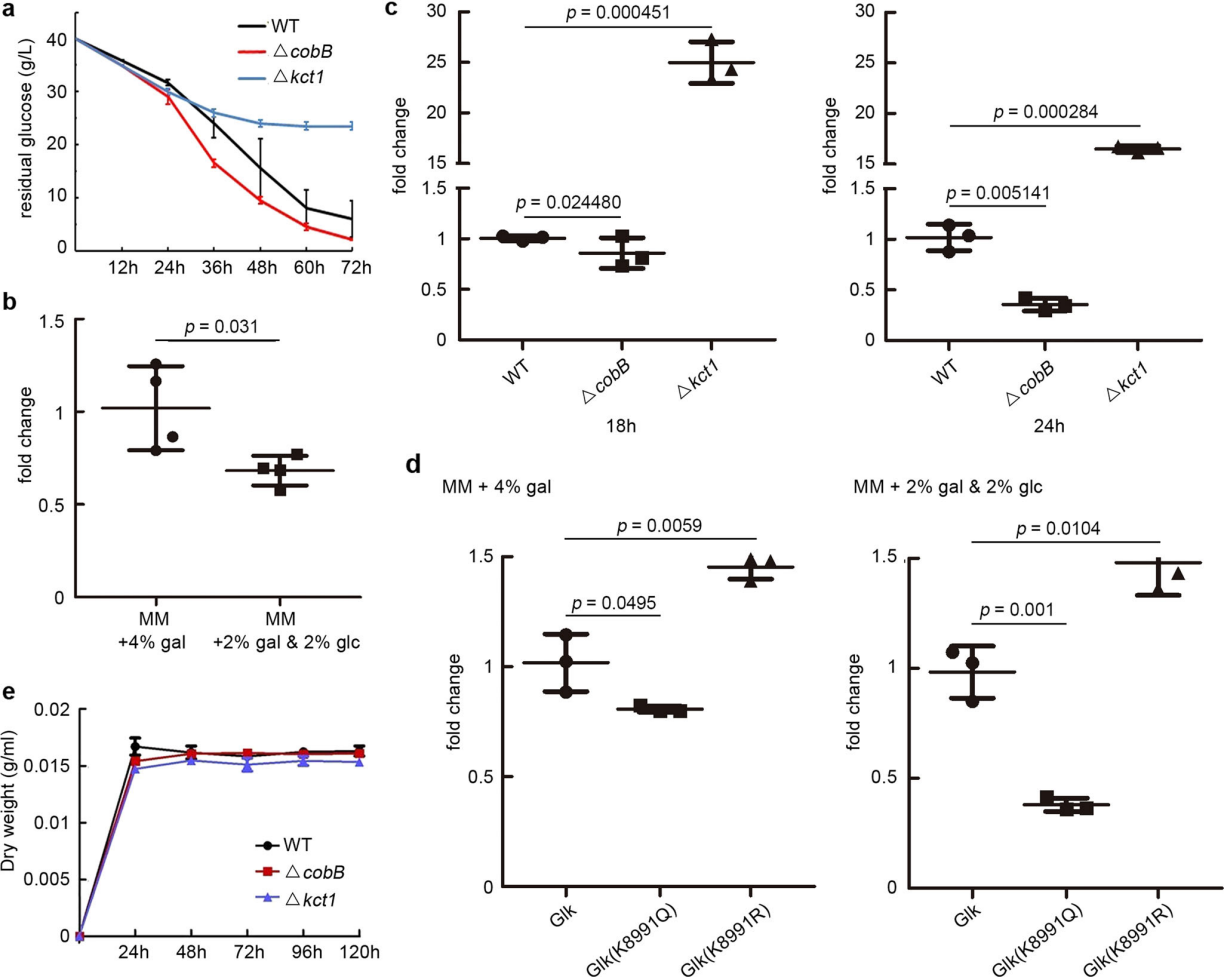
Crotonylation regulates carbon utilization in *S. roseosporus*. **a** Residual glucose in the medium of *S. roseosporus* wild type and *ΔcobB* and *Δkct1* mutants. Bacterial strains were cultured in the YEME medium, and glucose concentration was measured every 12 h. Experiments were performed in triplicate. **b** Quantitative assays of fold change of *galK* gene expression in *Δglk* mutant complemented with *glk*. Cells were cultured in MM media with different carbon sources. Gal galactose, glc glucose. Experiments were performed in quadruple. **c** Quantitative assays of fold change of *galK* gene expression in wild type and *ΔcobB* and *Δkct1* mutants. Cells were cultured in YEME medium with 4% glucose for 18 or 24 h. Experiments were performed in triplicate. **d** Quantitative assays of fold change of *galK* gene expression in *Δglk* complemented with *glk* and its mutants (K8991Q, K8991R). Cells were cultured in MM media with different carbon sources for 24 h. Gal galactose, glc glucose. Experiments were performed in triplicate. **e** Dry weight of wild type and *ΔcobB* and *Δkct1* mutants in the YEME culture without glucose but with galactose as the sole carbon source.

Glucose has been shown to trigger CCR in *Streptomyces* for sugar metabolism, cell differentiation, and secondary metabolite production^52^. First, the residual glucose in the culture was examined. We found that glucose was consumed faster in the *ΔcobB* mutant (Fig. 8a), though the Glk activity was much lower (Fig. 7d), while the *Δkct1* mutant showed more glucose surplus than wild type (Fig. 8a). These data indicated that the ratio of glucose uptake/Glk kinase activity was highest in the *ΔcobB* mutant but lowest in the *Δkct1* mutant, suggesting that the *ΔcobB* mutant had the strongest CCR, while the *Δkct1* mutant had the weakest CCR, according to the index of glucose uptake/Glk activity ratio suggested previously^18^. In addition, considering that over-crotonylation of bacterial proteins is the direct consequence in the *ΔcobB* mutant, our data suggested that crotonylation should positively regulate CCR.

To further confirm this hypothesis, we investigated the utilization of non-fermentable carbon sources (herein galactose) in the *ΔcobB*, *Δkct1*, and *Δglk* mutants complemented with Glk or its point mutants to mimic the crotonylated form (K8991Q) or non-crotonylated form (K8991R). The transcription levels of *galK*, a gene encoding galactokinase^53^ for galactose utilization, were examined. We found repressed *galK* expression in the presence of glucose (Fig. 8b), but it was strongly de-repressed in *Δkct1* mutant (Fig. 8c) or with the non-crotonylated form of Glk (K8991R), even in the presence of glucose (Fig. 8d), suggesting loss of CCR controls. However, galK expression was further repressed in the *ΔcobB* mutant (Fig. 8c) or when Glk (K8991Q) mutant protein was complemented even in the absence of glucose (Fig. 8d), suggesting the strengthened CCR effects. These results suggested that crotonylation on K89/K91 of Glk is required for proper CCR, and removal of this PTM could constitutively release cells from CCR. Consistent with this hypothesis, the growth defect of *Δkct1* mutant, in which crotonylation of Glk apparently reduced, was rescued to a comparable level of wild type if galactose was the sole carbon source (Fig. 8e). All these data suggested that crotonylation positively regulates CCR in *Streptomyces*.

## Discussion

### Crotonylation of *Streptomyces* metabolic pathways and enzymes

Here, based on crotonyl-proteomics, we showed several characteristics of protein crotonylation in *Streptomyces*. First, *Streptomyces* proteins are highly posttranslationally modified as crotonylation. About 20% proteins and 4000 lysine sites in *S. roseosporus* are crotonylated, which is about 2-fold in protein and over 3-fold in the site of acetylation in the same species^10^. Considering that acetyl-CoA is the most abundant acyl-CoA within cells and estimated 1000-fold more than crotonyl-CoA^32^, our results suggested that crotonylation might play more broad and sensitive roles than acetylation in *Streptomyces*. Second, though crotonylated proteins are distributed in various cellular processes, such as cell cycle, growth, division, and cellular response to stimulus, most crotonylated proteins are enzymes with catalytic activities and involved in metabolic pathways, mainly in carbon metabolism and protein turn-over. For example, heavily crotonylated enzymes are enriched in glycolysis, pyruvate metabolism, TCA, phospho-pentose pathway, and CoA biogenesis, which are responsible for glucose catabolism, acetyl-CoA, and NAD(P)H production. These observations implied that crotonylation might regulate carbon utilization and biogenesis of acyl-CoA species, thus causing complex interplay of crotonylation with other acyl-CoAs both at intracellular concentrations and their possible competitive modifications. Moreover, almost all aminoacyl-tRNA synthetases, numerous ribosomal proteins and protein degradation machineries (proteases, proteasome) are also highly crotonylated, suggesting that crotonylation is one of the determinants for protein turn-over in cell metabolism regulation. Third but not the last, crotonylation is involved in secondary metabolism, including cell differentiation and secondary metabolite biosynthesis. This is characterized by modification of some transcriptional regulators for secondary metabolism development and the biosynthetic enzymes for secondary metabolite production.

### Crotonylation positively regulates CCR of *Streptomyces*

*Streptomyces* is soil dwelling and has to compete with other microbes in the living community for nutrients, such as the chitin-degradative product *N*-acetyl-glucosamine^54^. CCR has evolved in most microorganisms to use one carbon source in priority to support vegetative growth by suppressing the utilization of other carbon sources^13,55^. For *Streptomyces*, glucose has been extensively used in laboratories as the main carbon source in feast, and glucose-induced CCR has been shown to suppress bacterial differentiation, including morphogenesis and secondary metabolism development. Glk not only functions as a kinase to activate glucose in the first step of glucose catabolism but also has a regulatory role, proposed via a kind of PTM to interact with other transcriptional regulators, on CCR modulation^13,20^.

Here we found that all enzymes are crotonylated in glucose catabolism, including glycolysis, pyruvate dehydrogenesis, and TCA, suggesting that crotonylation is involved in regulation of glucose utilization and subsequent CCR. Indeed, Over-crotonylation in a decrotonylase mutant *ΔcobB* not only increases Glk modification to reduce its kinase activity but also promotes total glucose consumption, suggesting that heavy crotonylation leads to high ratio of glucose uptake/Glk activity. Moreover, over-crotonylation suppresses the utilization of non-preferred carbon sources, indicating the intensified CCR. These observations suggested that high crotonylation will lead to CCR. We suspected that crotonylation might be one of the PTMs for Glk to interact with regulators to trigger CCR. So here we proposed that crotonylation modulates *Streptomyces* metabolism at least by positive regulation of CCR through dual regulation of Glk and carbon source metabolism. But the mechanism how crotonylation regulates interaction of Glk with other regulators still needs further investigation.

### Crotonylation dynamics in regulation of *Streptomyces* metabolism

In this work, we showed that crotonylation during *Streptomyces* metabolism is properly controlled by a set of modification enzymes, including two decrotonylases CobB, HDAC, and one crotonyl-transferase Kct1. They play their roles in different development phases, as CobB mainly erases crotonylation during secondary metabolism, while HDAC and Kct1 work in the early stages. Abnormal crotonylation in these mutants results in altered patterns of cell growth and metabolite production, suggesting that the concerted crotonylation is essential for proper metabolism of *Streptomyces*.

Based on our understanding of crotonylation on regulation of CCR, we propose that gradually increasing modification of *Streptomyces* proteins can maintain CCR for continuous preferred carbon source (such as glucose as a model in the laboratory cultivation) utilization and vegetative growth. Since glucose drops rapidly in the medium in the early phases and loses its control on CCR, crotonylation can reinforce CCR to make sure that *Streptomyces* can keep consuming glucose in priority. This hypothesis is supported by the observation that the *Δkct1* mutant has reduced cell growth accompanied by weakened CCR, as characterized by reduced glucose utilization and hyper-expression of genes for utilization of galactose. However, after vegetative growth, crotonylation needs to be partially erased to relieve CCR or reduce the modification of some regulators or biosynthetic enzymes for proper secondary metabolism development and efficient natural product biosynthesis, as block in hyper-crotonylation in the *ΔcobB* mutant is detrimental to secondary metabolite production.

In summary, here we showed that crotonylation, a previously unreported protein PTM, comprehensively regulates the metabolism of *Streptomyces*. Specifically, crotonylation positively regulates CCR for the proper development of *Streptomyces* metabolism. Our findings provide insights of PTM regulatory mechanisms on *Streptomyces* metabolism so far and the basis for possible molecular engineering of this modification system to take the full advantage of this genus for natural product development.

## Methods

### Strains, media, and biological materials

All strains used in this study are listed in Table 1. Wild-type *S. roseosporus* strain L30 was a daptomycin producer^40^. All the *S. roseosporus* mutants (*ΔprcB/A*, *ΔcobB*, *Δhdac*, *Δkct1*) were constructed based on the temperature-sensitive plasmid pKC1139^40^. The plasmids were transferred into wild-type strain by conjugation^4^. After double cross-over, the genotypes were verified by PCR. *E. coli* DH5α (Novagen) was the host for plasmid construction, and ET12567/pUZ8002 was used for conjugation to introduce DNA from *E. coli* to *S. roseosporus*^40^. Proteins were expressed and purified from *E. coli* BL21 (DE3) (Novagen).

**Table 1:** *Streptomyces* strains used in this study.

Strain	Description	Source or references
Wild type	*Streptomyces roseosporus* L30	^40^
*ΔprcB/A*	In-frame deletion of *prcB/A* in wild type	This work
*ΔcobB*	In-frame deletion of *cobB* in wild type	This work
*Δhdac*	In-frame deletion of *hdac* in wild type	This work
*Δkct1*	In-frame deletion of *kct1* in wild type	This work
*Δglk*	In-frame deletion of *glk* in wild type	This work
*Δglk*:: pSN7-*glk*	Glk tagged with FLAG at N terminus, overexpressed under ermEp* in *Δglk*	This work
*Δglk*:: pSN7-*glk*(K8991Q)	Glk(K8991Q) tagged with FLAG at N terminus, overexpressed under ermEp* in *Δglk*	This work
*Δglk*:: pSN7-*glk*(K8991R)	Glk(K8991R) tagged with FLAG at N terminus, overexpressed under ermEp* in *Δglk*	This work
WT::pSN7-*glk*	Glk tagged with FLAG at N terminus and His at C terminus, overexpressed under ermEp* in wild type	This work
*ΔcobB*::pSN7-*glk*	Glk tagged with FLAG at N terminus and His at C terminus, overexpressed under ermEp* in *ΔcobB*	This work
*Δkct1*::pSN7-*glk*	Glk tagged with FLAG at N terminus and His at C terminus, overexpressed under ermEp* in *Δkct1*	This work
*S. coelicolor* M145	Wild type, SCP1^−^, SCP2^−^	^33^
*S. lividans* TK24	Wild type	^34^
*S. albus* J1074	Wild type	^35^
*S. tsukubaensis* L19	Wild type	^36^

Liquid tryptic soy broth with 5% PEG6000 was the media for *S. roseosporus* mycelium preparation, and YEME (0.3% yeast extract, 0.3% malt extract, 0.5% tryptone, 4% glucose) was used for daptomycin production. R5 solid medium worked for sporulation, while MS solid medium was used for conjugation. All *E. coli* strains were cultured in LB medium^4^. Minimum medium (MM) (0.2% NH_4_Cl, 0.1% (NH_4_)_2_SO_4_, 0.05% KCl, 0.05% NaCl, 0.1% KH_2_PO_4_, 0.05% MgSO_4_•7H_2_O, 0.002% FeSO_4_•7H_2_O) was used for quantificational detection of transcription of related genes for other carbon utilization.

### Plasmid construction

All plasmids and primers are provided in Table 2 and Supplementary Data 1, respectively. For construction of pKC1139-based plasmids for deletion, the left and right homologous fragments of *prcB/A*, *cobB*, *hdac*, *kct1*, and *glk* were amplified with primer pairs 1 + 2 and 3 + 4, 7 + 8 and 9 + 10, 13 + 14 and 15 + 16, 19 + 20 and 21 + 22, and 161 + 162 and 163 + 164 respectively, and sequentially recombined to pKC1139 digested with *Xba*I/*Bam*HI by the ClonExpress MultiS One Step Cloning Kit (Vazyme, China). pET28a and pET32a vectors were used for protein expression in *E. coli*. The DNA fragment of *glk* was amplified with primer pair 25 + 26 and cloned into the *Eco*RI/*Bam*HI site of pET32a to yield the plasmid pET32a-*glk*. The fragments of *cobB* (primers 33 + 34) and *kct1* (primers 35 + 36) were amplified and inserted into the *Nde*I/*Bam*HI site of pET28a by the ClonExpress II One Step Cloning Kit (Vazyme). The expression vectors of Glk mutants were amplified from the plasmids pET32a-*glk* using primers 27–32 by PCR and confirmed by DNA sequencing. The fragment used for Bacterial Two-Hybrid System of *glk* (primers 39 + 40) was recombined to pUT18 digested with *Xba*I/*Bam*HI. *cobB* (primers 41 + 42) and *kct1* (primers 43 + 44) were recombined to pKT25 digested with *Xba*I/*Bam*HI by the ClonExpress II One Step Cloning Kit (Vazyme). *glk* was amplified with primer pair 37 + 38 and cloned into *Xb*aI/*Bgl*II site of pSN7^37^ by the ClonExpress II One Step Cloning Kit (Vazyme). All the fragments were amplified with KOD-Plus-Neo (Toyobo) using *S. roseosporus* L30 genomic DNA as the template.

**Table 2:** Plasmids used in this study.

Plasmid	Description	References
pKC1139	Bifunctional *oriT* RK2 plasmid for gene deletion	^40^
pKC1139-*ΔprcB/A*	*prcB/A* knockout plasmid based on pKC1139	This work
pKC1139-*ΔcobB*	*cobB* knockout plasmid based on pKC1139	This work
pKC1139-*Δhdac*	*hdac* knockout plasmid based on pKC1139	This work
pKC1139-*Δkct1*	*kct1* knockout plasmid based on pKC1139	This work
pKC1139-*Δglk*	*glk* knockout plasmid based on pKC1139	This work
pET32a	Expression vector in *E. coli* with His tag	Novagen
pET32a-*glk*	*glk* in pET32a	This work
pET32a-*glk* (K89Q)	*glk* (K89Q) in pET32a	This work
pET32a-*glk* (K91Q)	*glk* (K91Q) in pET32a	This work
pET32a-*glk* (K8991Q)	*glk* (K8991Q) in pET32a	This work
pET28a	Expression vector in *E. coli* with His-tag	Novagen
pET28a-*cobB*	*cobB* in pET28a	This work
pET28a-*kct1*	*kct1* in pET28a	This work
pUT18	Vector for bacterial two-hybrid system	This work
pKT25	Vector for bacterial two-hybrid system	Euromedex
pUT18-*zip*	Control plasmid for bacterial two-hybrid system	Euromedex
pKT25-*zip*	Control plasmid for bacterial two-hybrid system	Euromedex
pUT18-*glk*	*glk* in pUT18	Euromedex
pKT25-*cobB*	*cobB* in pKT25	This work
pKT25-*kct1*	*kct1* in pKT25	This work
pSN7	Overexpression integrative shuttle vector containing ermEp* with 3×FLAG tag at N terminus and 18×His tag at C terminus	^37^
pSN7-*glk*	*glk* in pSN7	This work
pSN7-*glk* (K8991Q)	*glk* (K8991Q) in pSN7	This work
pSN7-*glk* (K8991R)	*glk* (K8991R) in pSN7	This work

### Crotonyl-proteomic analysis of *Streptomyces* proteins

Identification of crotonylated proteins and the following in silico analysis were demonstrated by PTM Biolabs Inc. (Hangzhou, China). Detailed procedures are described in Supplementary Materials and Methods.

### GO and KEGG analysis

GO annotation is a bioinformatics analysis method that can organically link various information of genes and gene products (such as proteins) to provide statistical information. GO annotations of proteome were derived from the UniProt-GOA database (http://www.ebi.ac.uk/GOA/). First, the system would convert the protein ID to UniProt ID, which would be used to match the GO ID. Next we retrieved the corresponding information from the UniProt-GOA database according to the GO ID. If there was no protein information in the UniProt-GOA database, an algorithm software based on protein sequences, InterProScan, would be used to predict the GO function of the protein. This protein was then classified according to cellular composition, molecular function, or physiological process. Then we used the KEGG pathway database to annotate protein pathways. First, we used the KEGG online service tool KAAS to annotate the submitted proteins and then used KEGG mapper to match the annotated proteins to the corresponding pathways in the database.

### Construction of bacterial two-hybrid library

Fifty-four putative acyl-transferases and two de-acylases in two-hybrid library are listed in Supplementary Data 6. All fragments used for this library construction were recombined to *Xba*I/*Bam*HI-digested pKT25 by ClonExpress II One Step Cloning Kit (Vazyme), and primers used are listed in Supplementary Data 1. Plasmids were transformed individually into *E. coli* BTH101, which was growth in LB media with kanamycin. A single colony from each plate was cultured in LB in 96-well clear flat bottom polystyrene microplates (Corning), mixed together, and saved in ultra-low temperature after overnight culture at 37 °C.

### Metabolite analysis

The metabolites pyruvate and ATP were determined with the Pyruvate Assay Kit (Solarbio, BC2205) and ATP Assay Kit (Beyotime, S0026), respectively, according to the manufacturer’s instructions. Daptomycin was analyzed as described previously^40^.

### Glk activity assays

Wild-type Glk and its mutant isoforms were expressed and purified from *E. coli* as previously described^40^. Glk activity assays were performed at 30 °C for 10 min in Buffer A (20 mM Tris-HCl pH 8.0, 100 mM KCl, 7.5 mM MgCl_2_, 2 mM DTT, 1 mM NAD^+^, 5 mM ATP, 0.1 M glucose, 0.4 U of glucose-6-phosphate dehydrogenase (Sangon Biotech, A003992)), and the signals were determined by spectrophotometer at 340 nm. For analysis of Glk from *Streptomyces* lysate, 100 µg of cell extract was added to the reactions along with 1 mM phenylmethylsulfonyl fluoride (PMSF). For activity assays of Glk from *E. coli*, the reaction was carried out with 10 µg of Glk.

### In vitro crotonylation and decrotonylation assays

Bacterial two hybrids for protein–protein interaction were performed with the Bacterial Adenylate Cyclase Two-Hybrid System Kit (Euromedex) according to the product manual. For in vitro crotonylation reaction, 1 µg of Kct1, 2 µg of Glk, and 10 mM of crotonyl-CoA (Sigma) were added in the reaction buffer (20 mM Tris-HCI (pH 8.0), 100 mM KCl, 7.5 mM MgCl_2_) and incubated at 30 °C for 1.5 h, followed by Glk activity assays or loading for sodium dodecyl sulfate-polyacrylamide gel electrophoresis (SDS-PAGE). Decrotonylation assays were performed in the same reaction conditions, but 2.5 µg of Glk + 2 µg of CobB were used. The total loading protein and crotonylation were analyzed by western blot with antibodies against His-tag (Sigma) and crotonyllysine (Kcr) (PTM-502, PTM Biolabs Inc.) (1:2000), respectively.

### In vivo decrotonylation assays by immunoprecipitation

The mycelia were collected and lysed in pre-cold phosphate-buffered saline (PBS) buffer by ultra-sonication on ice. The remaining debris were removed by centrifugation at 12,000 rpm at 4 °C for 10 min. Twenty µl of anti-FLAG M2 affinity Gel (sigma, A2220) were added and incubated for 3 h in a roller shaker. The resin was centrifuged for 2 min at 5000 × *g*, and the supernatant was removed. After wash with PBS buffer for three times, the immunoprecipitated complexes were boiled in 1× loading buffer (62.5 mM Tris-HCl (pH 6.8), 2% SDS, 10% (v/v) glycerol, and 0.002% bromphenol blue), subjected to SDS-PAGE, and immunoblotted with anti-Kcr (PTM-501, PTM Biolabs Inc.) or anti-FLAG (M2, Sigma) antibody (1:2000).

### RNA preparation and quantitative real-time PCR (qRT-PCR)

RNA was prepared from mycelia with the RN43-EASYspin Plus Kit (Aidlab Biotech Co., Ltd. China) according to the manufacturer’s protocol. Genomic DNA was removed with RNase-free DNase I (TaKaRa), and cDNA was prepared with MMLV (TakaRa) as described by the supplier. qRT-PCR was performed with SYBR Premix Ex Taq II (TaKaRa) for genes *hrdB*, *dptE*, and *galK* with primer pairs 45 + 46, 47 + 48, and 49 + 50, respectively. The relative fold changes of gene expression were calculated based on the formula of 2^–ΔΔCt^.

### LC-MS analysis of crotonyl-CoA and acetyl-CoA

Mycelia were collected and lysed in PBS supplemented with 1 mM PMSF. The remaining debris was removed by centrifugation at 10,000 rpm at 4 °C for 5 min, and the protein was precipitated with cold 20% TCA for 2 h at −20 °C. After centrifugation at 10,000 rpm at 4 °C for 10 min, the supernatant was transferred to a new centrifuge tube. Crotonyl-CoA and acetyl-CoA was further purified with an Oasis PRiME HLB 96-well Plate and 30 mg of Sorbent per Well (Waters, 186008054) according to the manufacturer’s instructions. Finally, effluent was concentrated on the CentriVap vacuum (Labconco) and subjected to LC-MS on an Agilent Eclipse TC-C18 column (5 μm, 4.6×250 mm, Agilent Technologies), at a flow rate of 0.5 ml/min. Solution A (water containing 20 mM ammonium acetate, pH = 7.4) and solution B (methanol with 20 mM ammonium acetate) were employed to isolate the components with ultraviolet detection set at 254 nm. Elution was initiated with constant 25% solvent B for 5 min and then performed with a linear gradient from 25% to 100% of solvent B in 20 min, followed by 100% solvent B for 5 min and re-equilibration to initial conditions for 5 min. The MS system was operated with an electrospray ionization source in positive ion mode^32^.

### Statistics and reproducibility

All statistics underlying the graphs and charts were analyzed by Excel 2017 and Prism (version 5, GraphPad). All experiments were independently performed in triplicate at least, indicated in the corresponding figure legend. Images were combined and annotated in Adobe Photoshop (version 2019).

### Reporting summary

Further information on research design is available in the Nature Research Reporting Summary linked to this article.

## Supplementary information

Supplementary Information

Description of Additional Supplementary Files

Supplementary Data 1

Supplementary Data 2

Supplementary Data 3

Supplementary Data 4

Supplementary Data 5

Supplementary Data 6

Supplementary Data 7

Reporting Summary

## Data Availability

All data supporting the findings of the current study are available within the article and Supplementary Information or from the corresponding authors upon request. Full blots and the mass spectrometric data for the modification peptides and residues are provided in Supplementary Information. The source data underlying plots shown in figures are shown in Supplementary Data 7.
