# Supplementary material for: Crotonylation of key metabolic enzymes regulates carbon catabolite repression in Streptomyces roseosporus
Source: Commun Biol. 2020 Apr 24;3:192. doi: 10.1038/s42003-020-0924-2 (PMC7181814; doi:10.1038/s42003-020-0924-2)
Supplement: Supplementary file 1 — Supplementary Information [file 42003_2020_924_MOESM1_ESM.pdf]

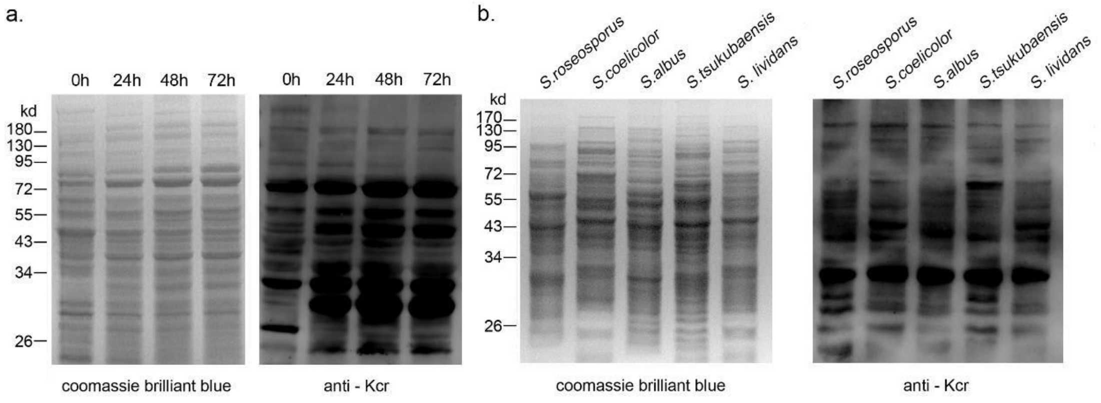

Supplementary Figure 1. Immuno-blot assays of total proteins from *Streptomyces* species.

20 μg of total protein were loaded, and the crotonylated proteins were detected with anti-Kcr monoclonal antibody while coomassie brilliant blue staining was used for the loading control.

a. Immuno-blot assay of total proteins from mycelia of *S. coelicolor* M145 with the anti-crotonylation antibody. Spores were inoculated from TSB medium and further cultured in liquid R5 medium for the times indicated.

b. Immuno-blot assay with the anti-crotonylation (anti-Kcr) antibody of whole-cell lysates from mycelia of *S. roseosporus*, *S. coelicolor*, *S. albus*, *S. tsukubaensis* and *S. lividans* cultured in TSB medium for how 24h

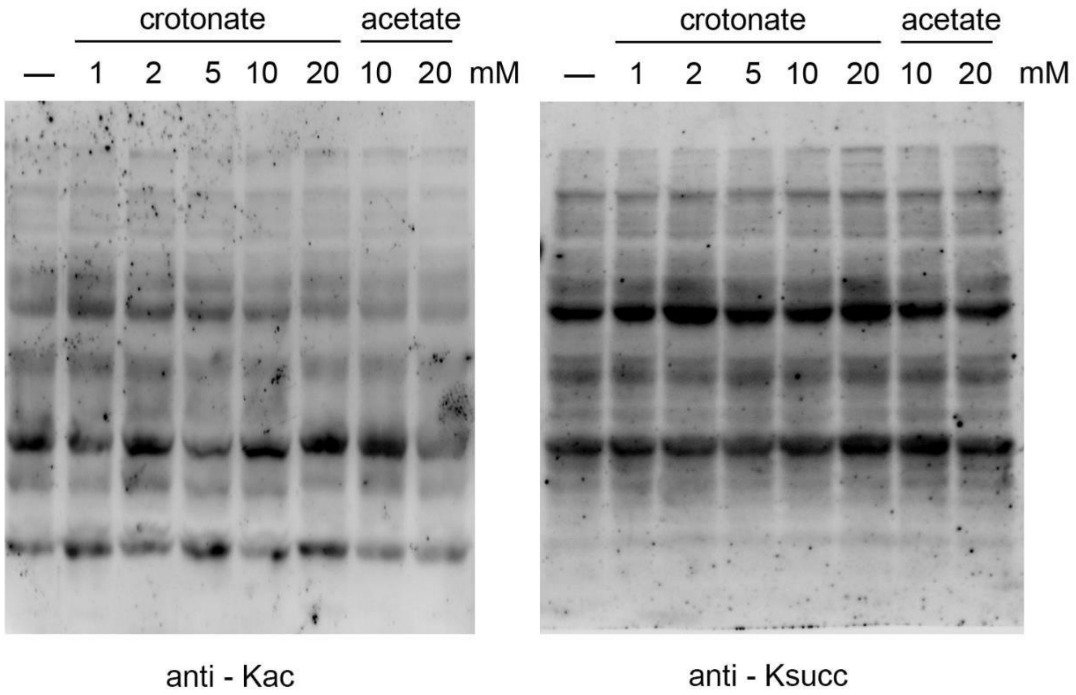

Supplementary Figure 2. Immuno-blot assays of *S. roseosporus* L30 with anti-acetylation (Kac) or succinylation (Ksucc) antibody of the global acetylation and succinylation in response to crotonate and acetate.

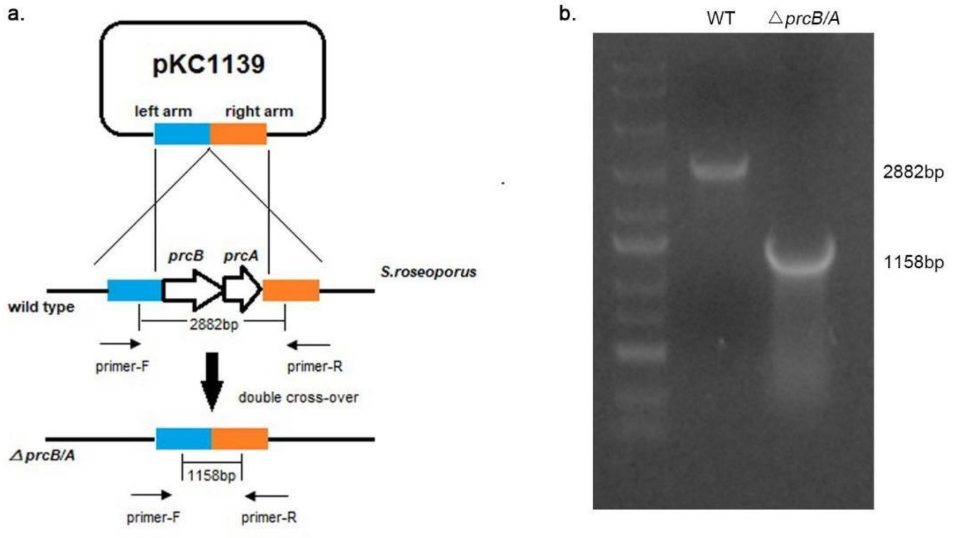

Supplementary Figure 3. In-frame deletion of *prcB/A* in *S. roseosporus* L30.

a. Schematic diagram of *prcB/A* knock-out. The positions of primers for PCR were shown as arrows, and the expected DNA fragment sizes were shown.

b. Confirmative PCR for *prcB/A* deletion. Primer-F and R were used to amplify the 2.8 kb and 1.1 kb fragments from the genomic DNA of wild type and the Δ*prcB/A* mutant, respectively.

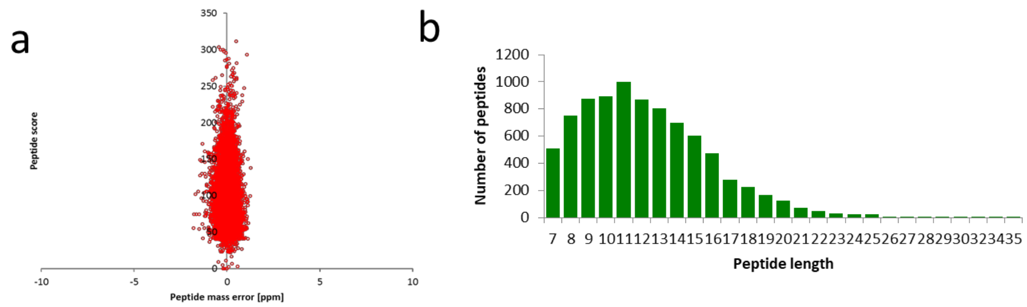

Supplementary Figure 4. Quality control of the MS/MS data.

All the crotonylated peptides identified have the mass error within 10 ppm (a), and most peptides have the length between 8-20 amino acids (b), which suggesting the mass error is less than 0.03 kD.

GO Terms Level 1	*GO Terms Level 2*	*No. of Protein*
Biological Process	metabolic process	672
	cellular process	511
	single-organism process	448
	biological regulation	110
	localization	81
	response to stimulus	71
	signaling	29
	cellular component organization or biogenesis	29
	other	2
Cellular Component	cell	170
	membrane	93
	macromolecular complex	77
	organelle	52
Molecular Function	catalytic activity	691
	binding	511
	transporter activity	55
	structural molecule activity	49
	nucleic acid binding transcription factor activity	30
	other	38

Supplementary Figure 5. Number of crotonylated proteins in Level 1 and Level 2 as shown from Gene Ontology (GO) analysis.

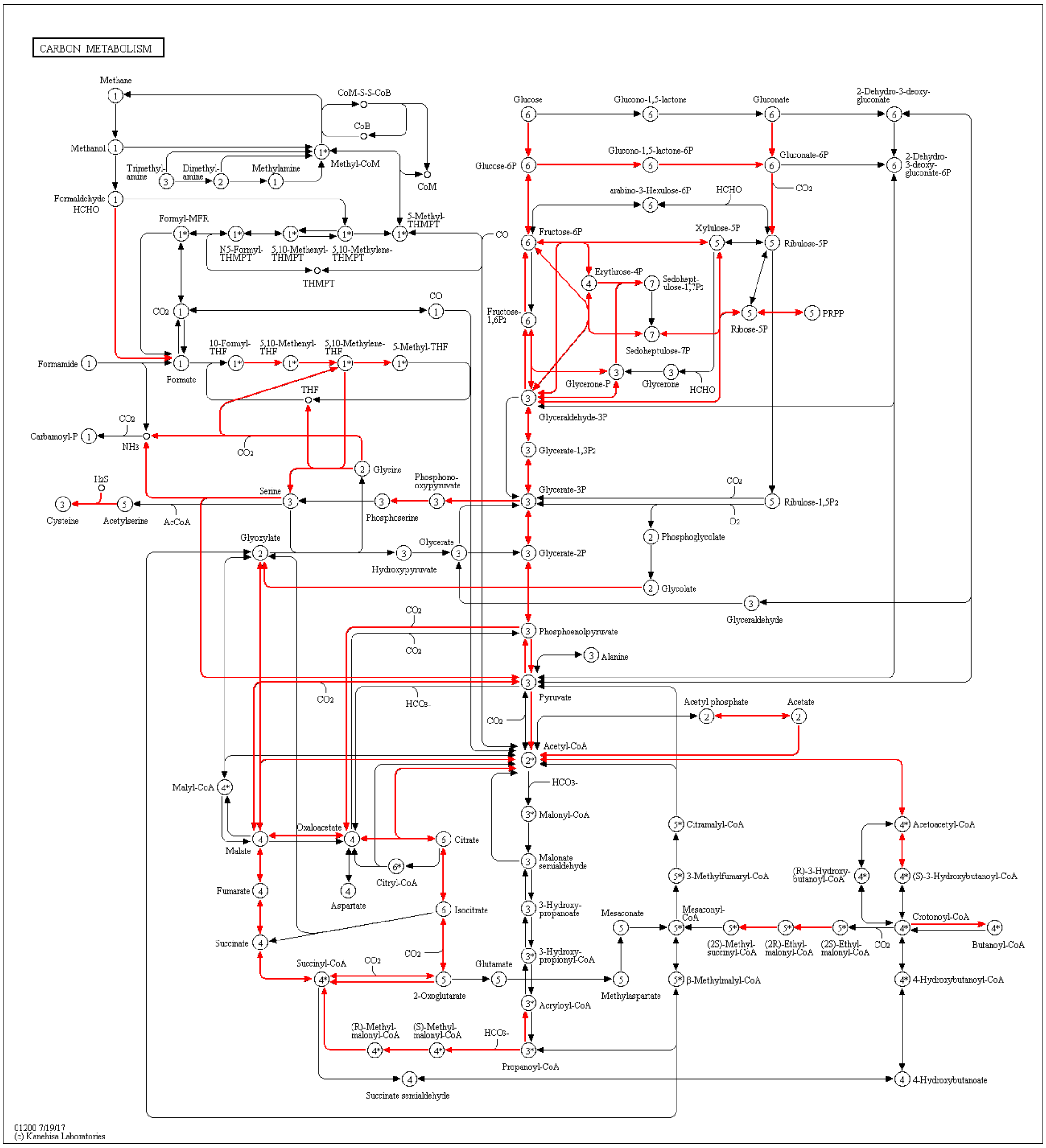

Supplementary Figure 6. KEGG analysis of enzyme overview carbon metabolism.

The crotonylated enzymes are in red arrows.

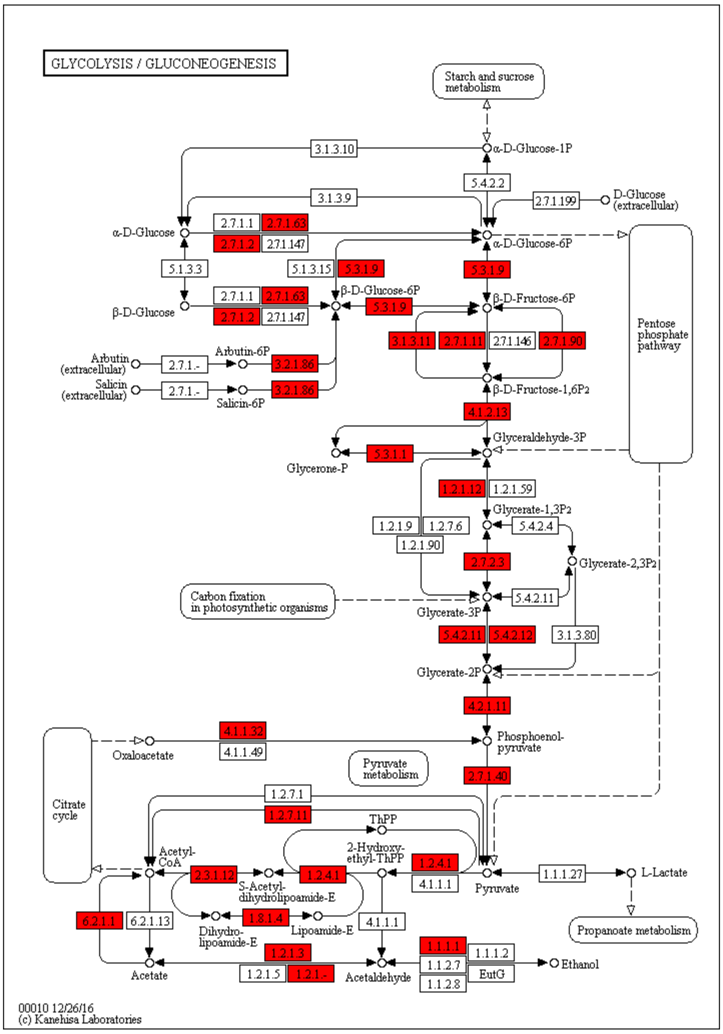

Supplementary Figure 7. KEGG analysis of enzymes involved in glycolysis and gluconeogenesis.

The crotonylated enzymes are in red.

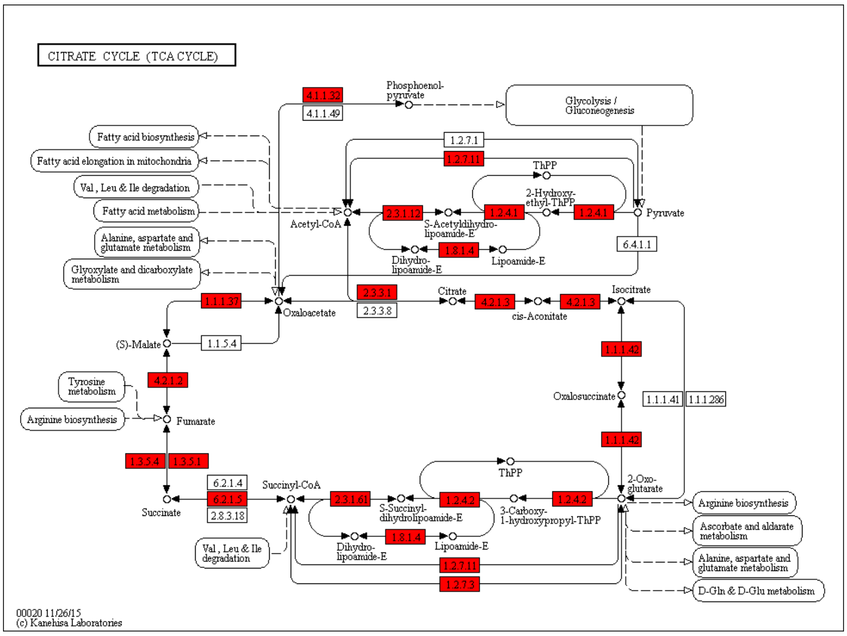

Supplementary Figure 8. KEGG analysis of enzymes involved in tricarboxylic acid cycle (TCA).

The crotonylated enzymes are in red.

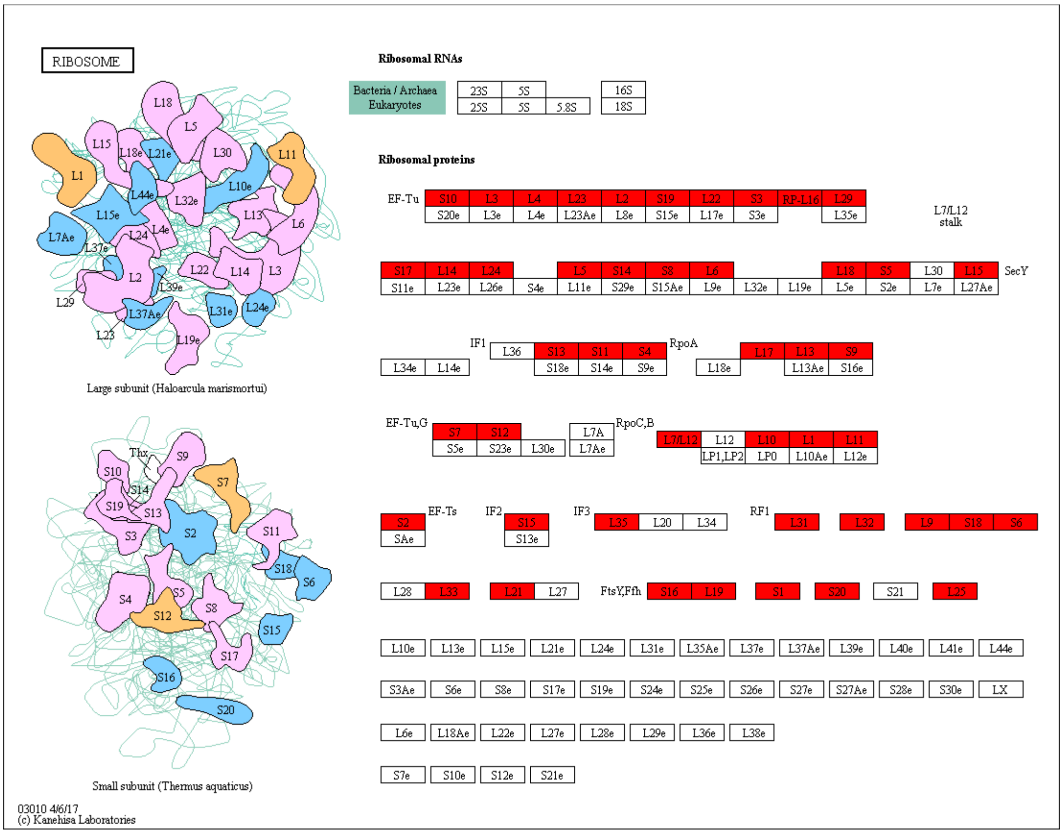

Supplementary Figure 9. KEGG analysis of ribosomal proteins.

The crotonylated proteins are in red.

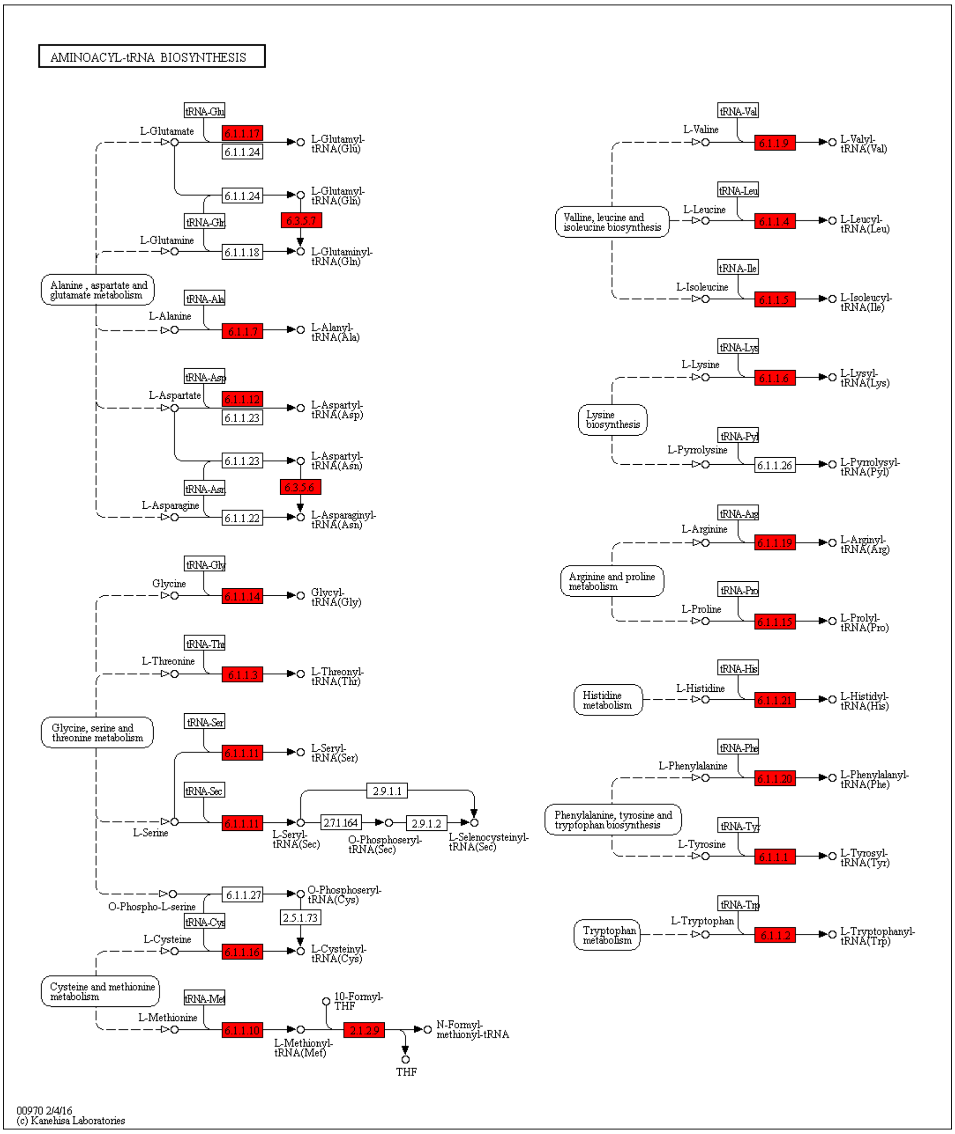

Supplementary Figure 10. KEGG analysis of aminoacyl-tRNA synthases.

The crotonylated enzymes are in red.

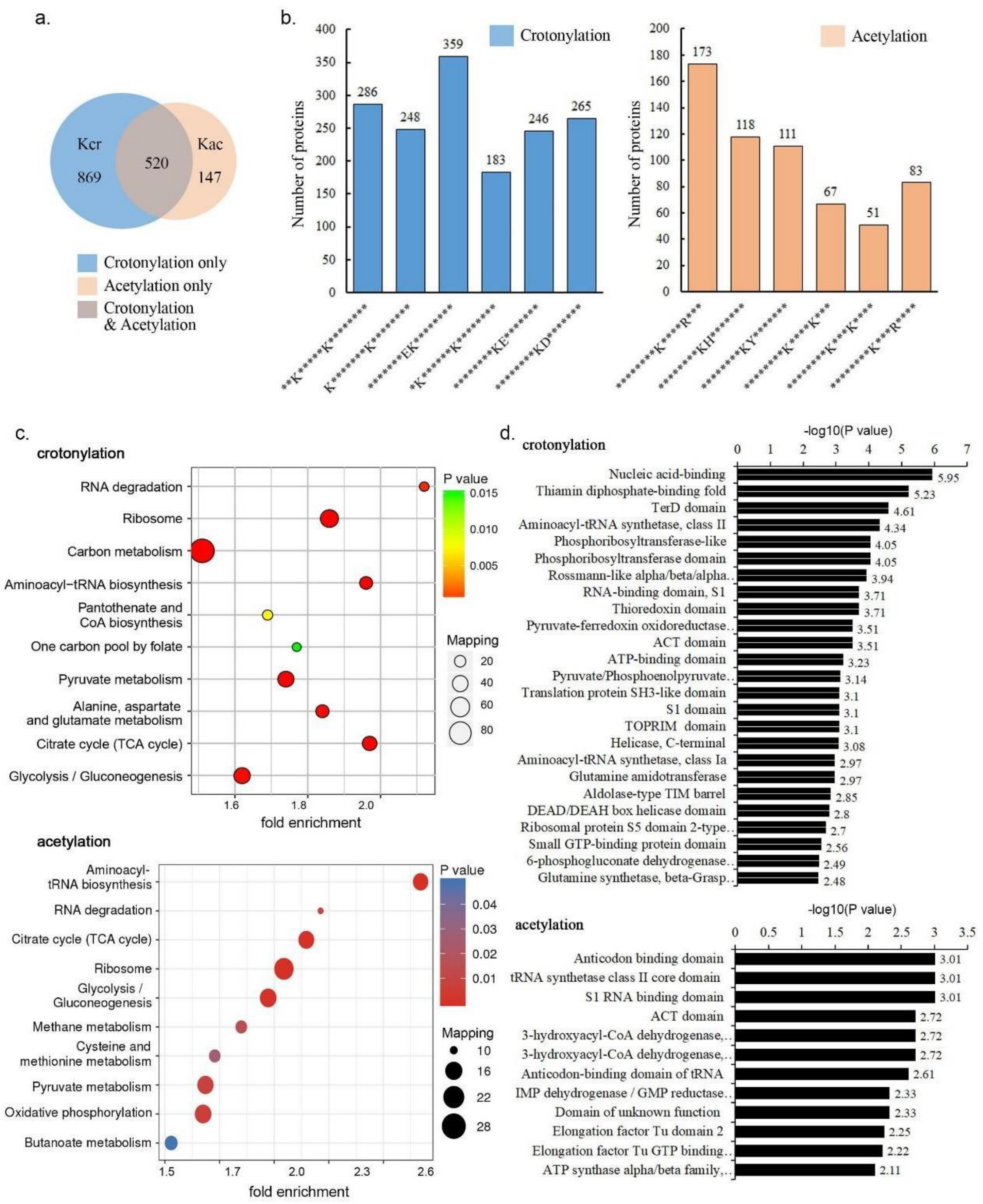

Supplementary Figure 11 Differences of substrate distribution, motif and pathway enrichment between acetylation and crotonylation profiles of *S. roseosporus*.

a. The overlap between crotonylated and acetylated substrates.

b. Statistical analysis of the significant motifs identified by Motif-X software.

c. and d. Enrichment analysis of the crotonylated proteins in *S.roseosporus*. KEGG pathway enrichment (c); and protein domain enrichment (d)

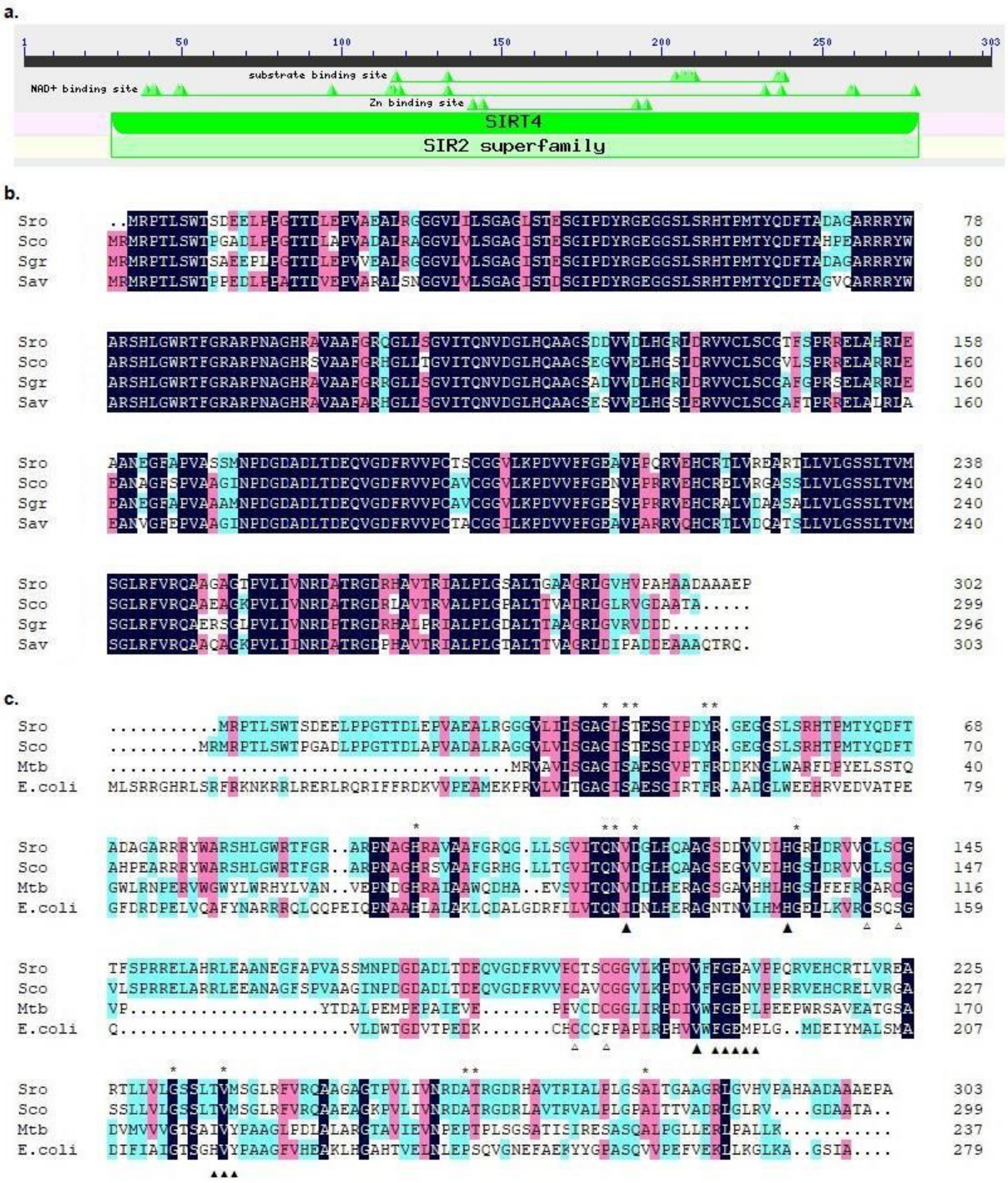

Supplementary Figure 12. Protein alignment of NAD^{+}-dependent deacetylase CobB (orf0088 in *S. roseosporus*), a Sir2 family regulator.

a. Putative conserved domains in CobB.

b. Alignment of amino acid sequences of the CobB in *S. coelicolor* (Sco), *S. avermitilis* (Sav), *S. griseus* (Sgr) and *S. roseosporus* (Sro).

c. Alignment of CobB from *S. coelicolor* (Sco), *S. roseosporus* (Sro), *M. tuberculosis* (Mtb) and *E. coli*. Residuals marked by * are NAD^{+} binding sites. △ means Zn^{2+} binding sites and ▲ means substrate binding sites.

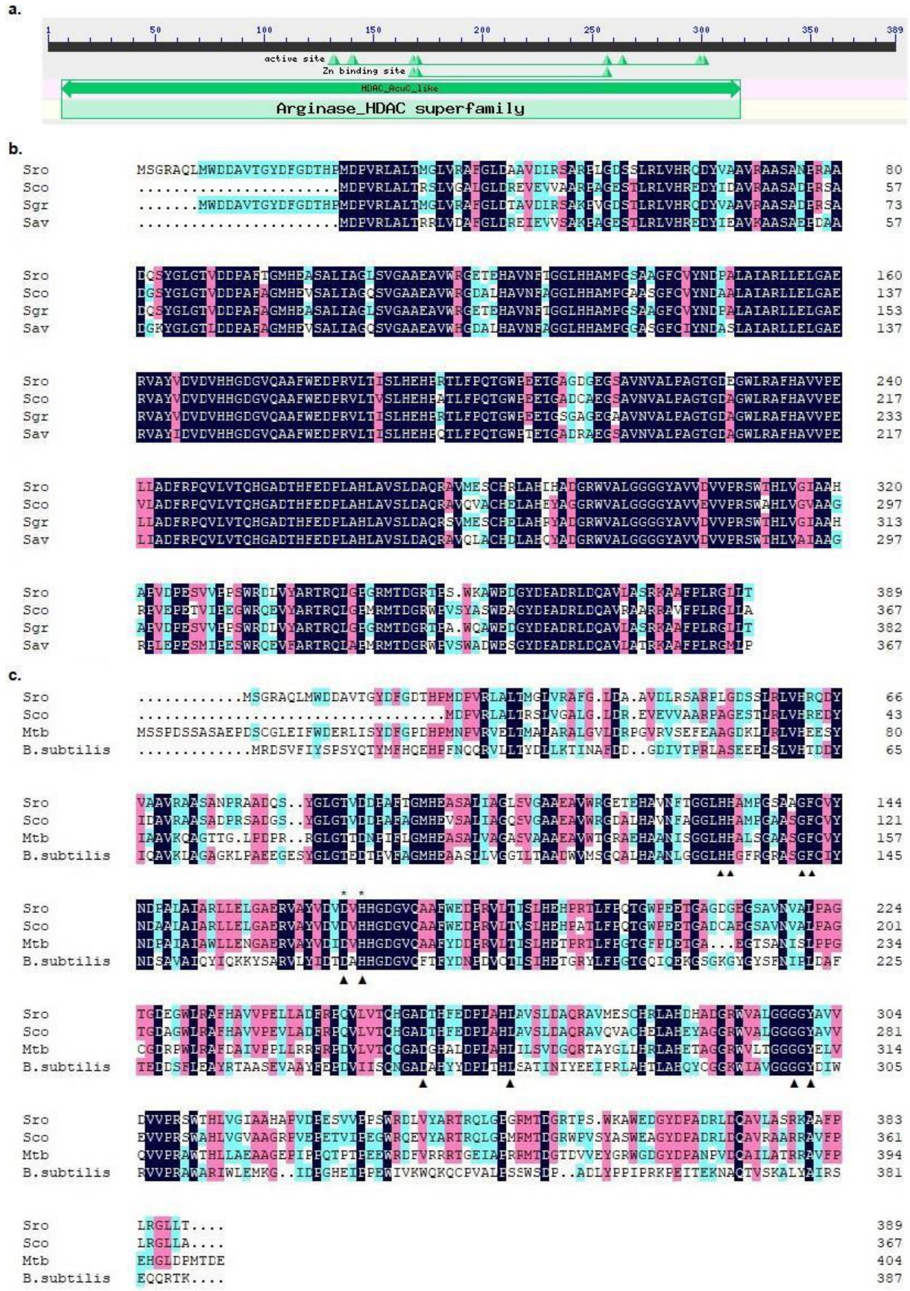

Supplementary Figure 13. Protein alignment of Zn^{2+}-dependent deacetylase HDAC (orf4227 from *S. roseosporus*).

a. Putative conserved domains in HDAC.

b. Alignment of HdaC from in *S. coelicolor* (Sco), *S. avermitilis* (Sav), *S. griseus* (Sgr) and *S. roseosporus* (Sro).

c. Alignment of HdaC from *S. coelicolor* (Sco), *S. roseosporus* (Sro), *M. tuberculosis* (Mtb) and *B. subtilis*. Residuals marked by * are Zn^{2+} binding sites and ▲ means active sites.

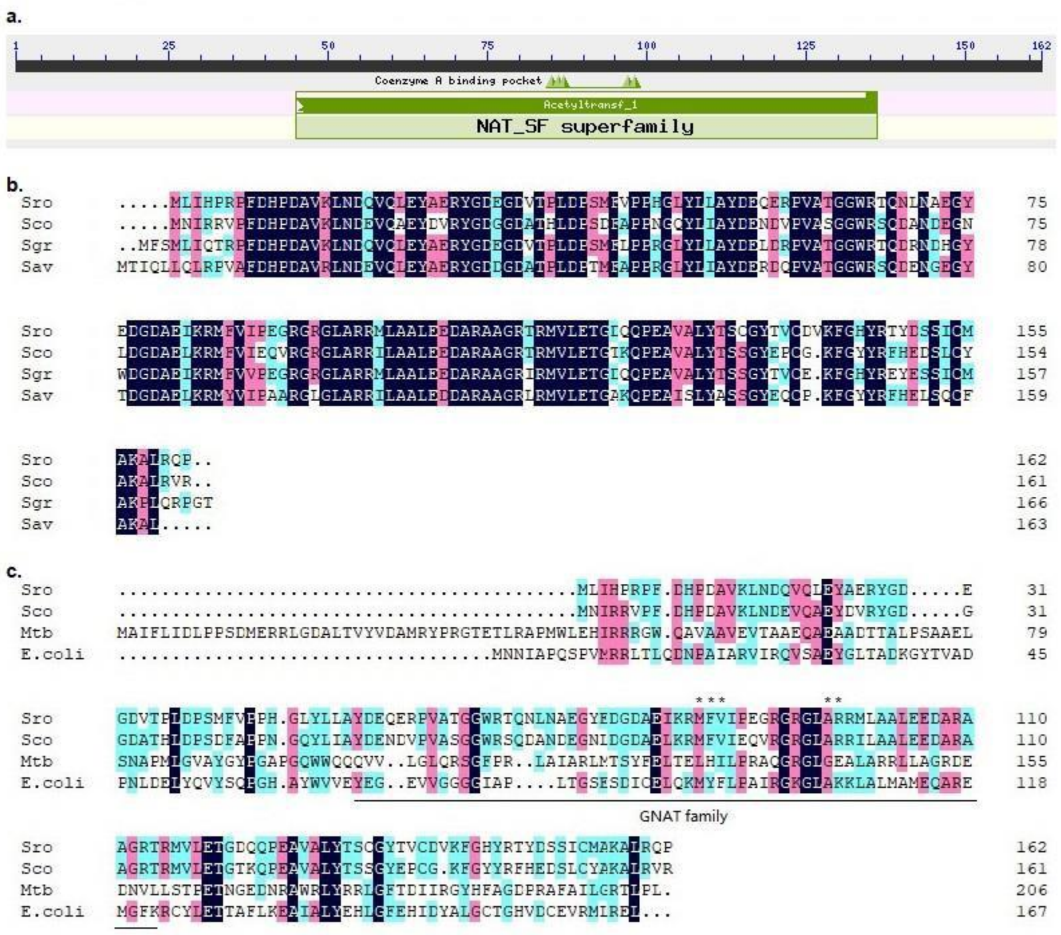

Supplementary Figure 14. Protein alignment of acetyltransferase Kct1 (orf4449 from *S. roseosporus*).

a. Putative conserved domains in Kct1.

b. Alignment of Kct1 from *S. coelicolor* (Sco), *S. roseosporus* (Sro), *M. tuberculosis* (Mtb) and *E. coli*. Residuals marked by * are CoA binding pocket, and the GNAT family motif is underlined

c. Alignment of Kct1 from *S. coelicolor* (Sco), *S. roseosporus* (Sro), *M. tuberculosis* (Mtb) and *E. coli*. Residuals marked by * are CoA binding pocket, and the GNAT family motif is underlined

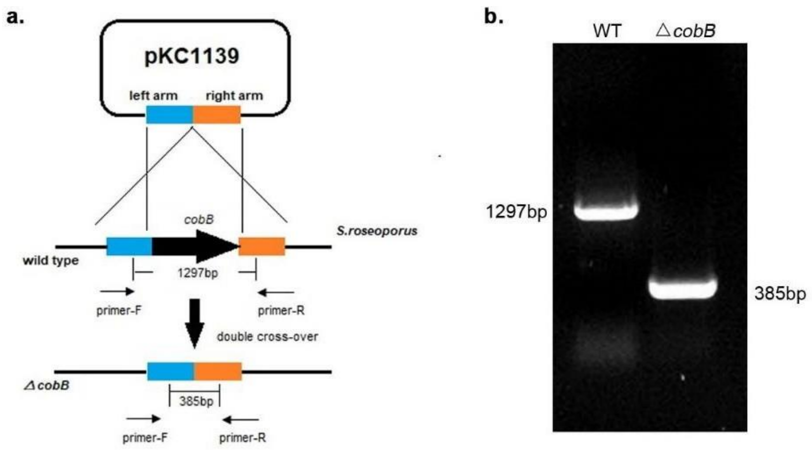

Supplementary Figure 15. In-frame deletion of *cobB* in *S. roseosporus* L30.

a. Schematic diagram of *cobB* knock-out. The positions of primers for PCR were shown as arrows, and the expected DNA fragment sizes were shown.

b. Confirmative PCR for *cobB* deletion.

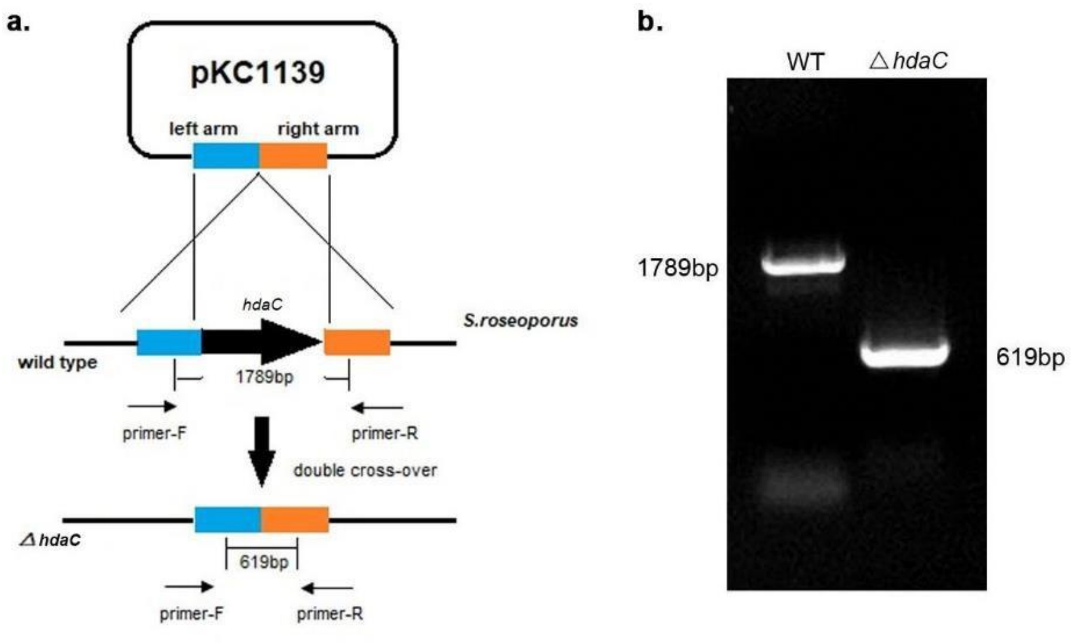

Supplementary Figure 16. In-frame deletion of *hdac* in *S. roseosporus* L30.

a. Schematic diagram of *hdac* knock-out. The positions of primers for PCR were shown as arrows, and the expected DNA fragment sizes were shown.

b. Confirmative PCR for *hdac* deletion.

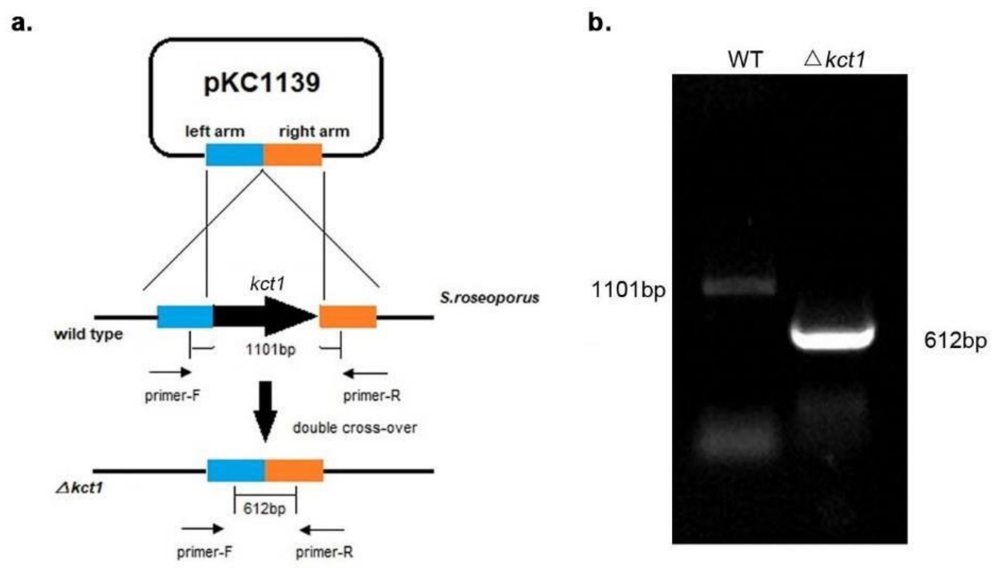

Supplementary Figure 17. In-frame deletion of kct1 in *S. roseosporus* L30.

a. Schematic diagram of *kct1* knock-out. The positions of primers for PCR were shown as arrows, and the expected DNA fragment sizes were shown.

b. Confirmative PCR for *kct1* deletion.

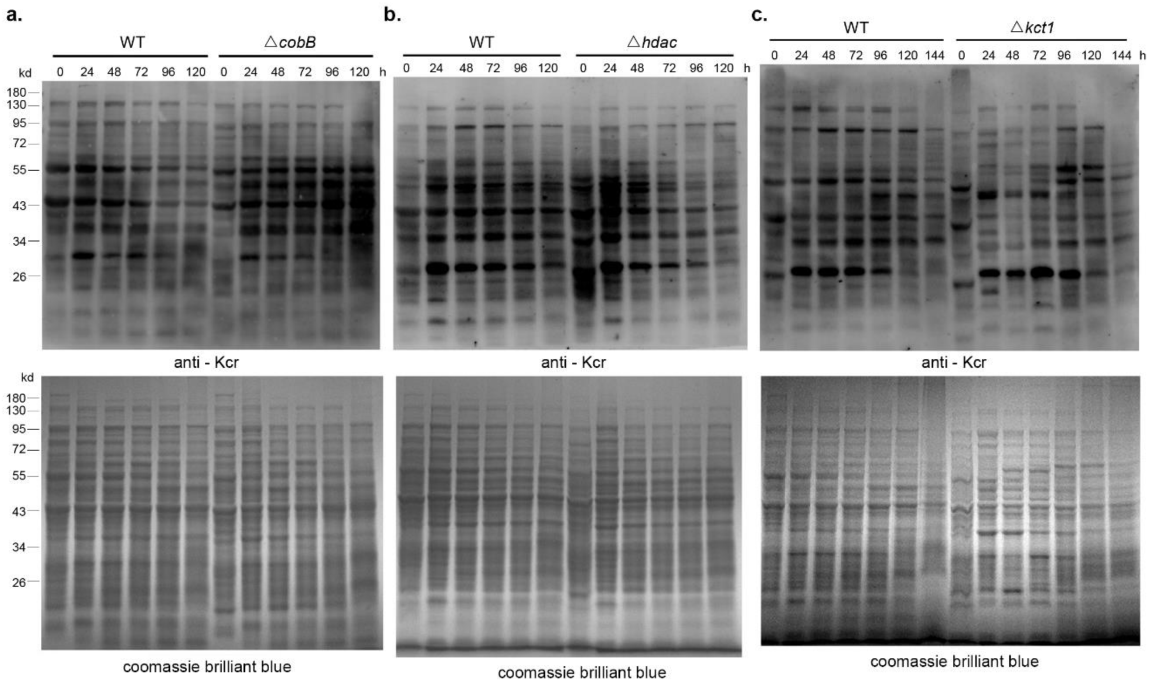

Supplementary Figure 18. Uncropped blot and gel images to identify crotonylation enzymes (relate to figure 4).

Immuno-blot assays of crotonylation from *S. roseosporus* strain wild type (WT), two decrotonylase mutants *ΔcobB* (a) and *Δhdac* (b), and a crotonyl-transferase mutant *Δkct1* (c) with anti-Kcr monoclonal antibody, together with coomassie blue staining for the loading control.

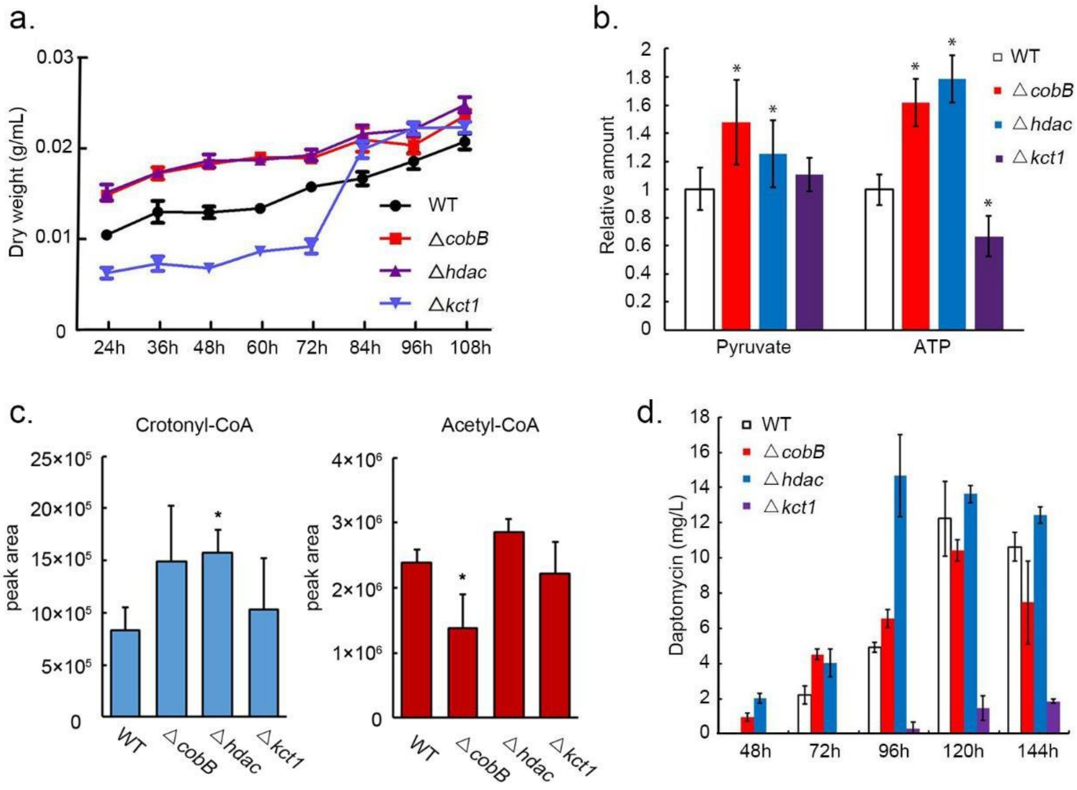

Supplementary Figure 19. Cell growth and metabolite production from *S. roseosporus* wild type and three mutants (*ΔcobB*, *Δhdac* and *Δkct1*).

a. Dry weight of *S. roseosporus* wild type and three mutants from fermentation in YEME medium. Samples were measured with 12 h intervals. The experiments were performed in triplicate.

b. Pyruvate and ATP levels of *S. roseosporus* wild type and three mutants cultured in YEME. Pyruvate and ATP were measured from the mycelia after 24 h, and *P* value was calculated with Student's t test (n = 3. *, $P < 0.05$). Values were shown as mean ± SD.

c. LC-MS analysis of cellular crotonyl-CoA and acetyl-CoA levels extracted from mycelia cultured in TSB for 24 h. The data represent mean peak area ± SD of three independent experiments. *P* value was calculated with Student's t test (*, $P < 0.05$)

d. Daptomycin production in wild type and three mutants. The daptomycin yield was measured for every 24 h. Experiments were performed in triplicate.

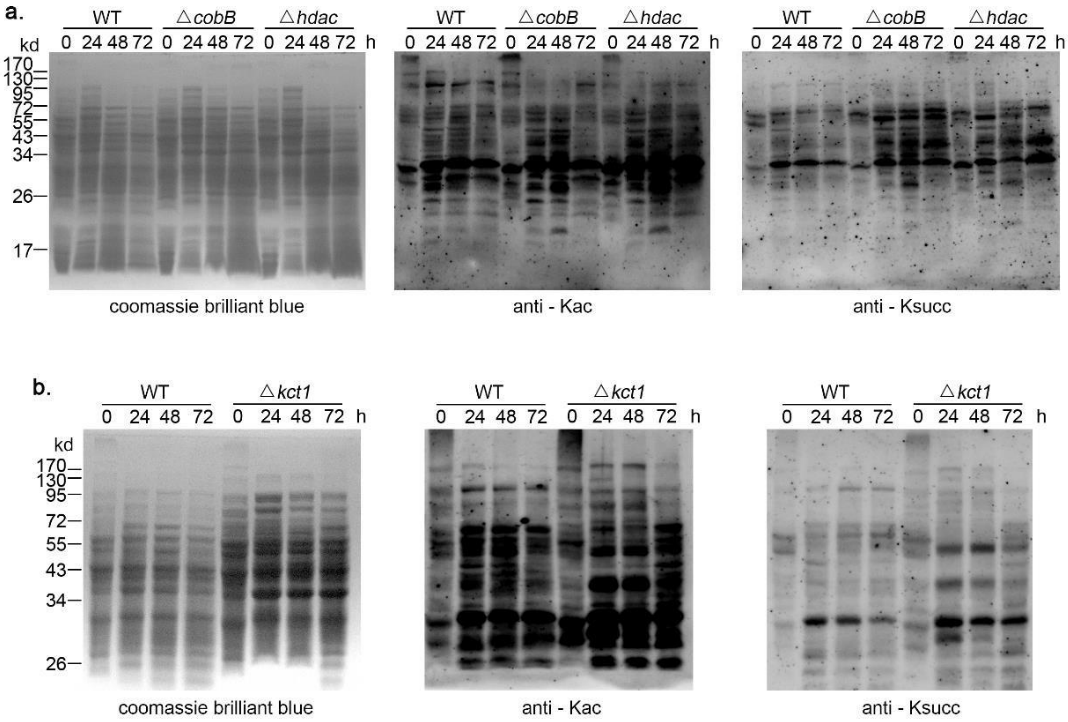

Supplementary Figure 20. Acetylation and succinylation profiles of *S. roseosporus* wild type and three mutants (*ΔcobB, Δhdac* and *Δkct1*).

a. Immuno-blots analysis of lysine acetylation and succinylation from protein lysates of *S. roseosporus* wild type, *ΔcobB* and *Δhdac* cultured in YEME medium.

b. Immuno-blots analysis of lysine acetylation and succinylation from protein lysates of *S. roseosporus* wild type and *Δkct1* mutant cultured in YEME medium.

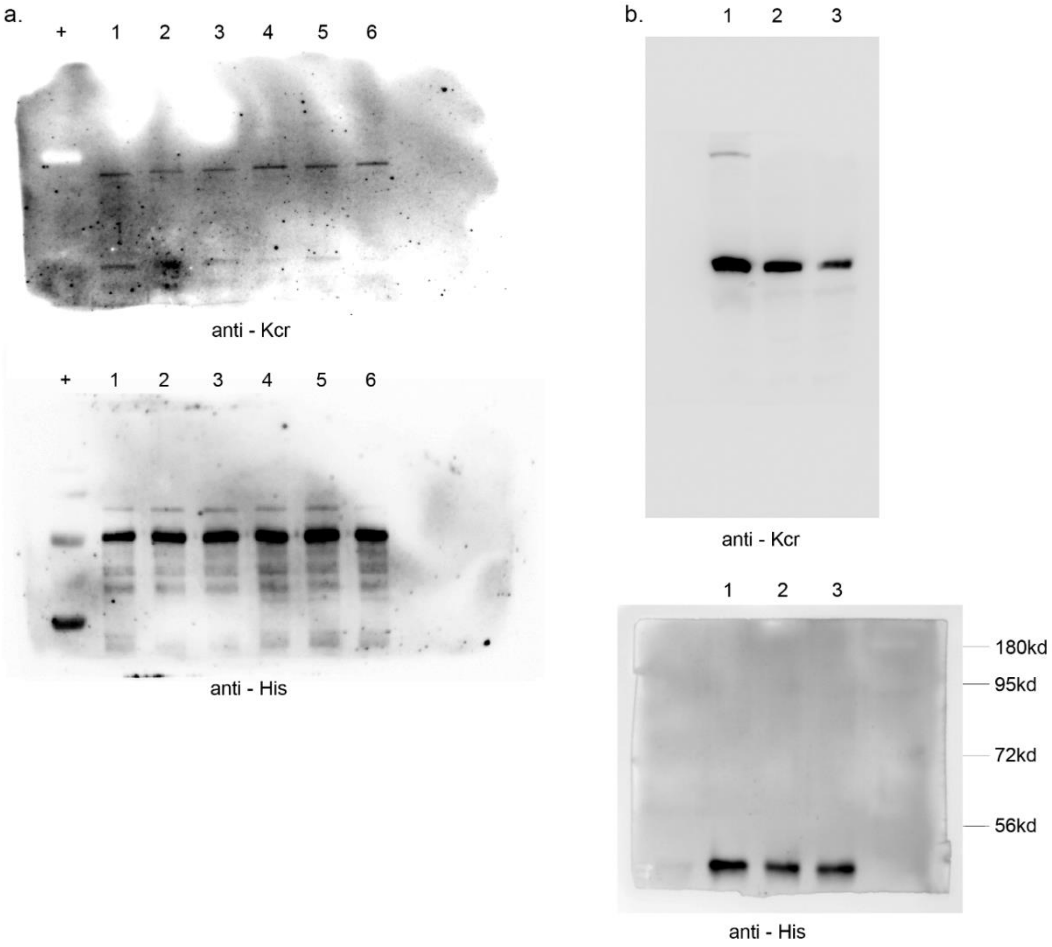

Supplementary Figure 21. Uncropped blot images of *in vitro* assays for Glk crotonylation and decrotonylation (relate to Figure 6).

a. Immuno-blots analysis of Glk crotonylation by Kct1 *in vitro*. The roles of Kct1 acting on Glk were testified by immunoblot with pan anti-Kcr monoclonal antibody, and we used immunoblots with anti-His monoclonal antibody as the loading controls of Glk. Lane + is a negative control with His tag about 49 kD protein purified from *E.coli* BL21. Lane 1 and 2 are only Glk used in this reaction. Lane 3 is Glk with 10 mM crotonyl-CoA, to testify nonenzymic reaction. Lane 4 - 6 are Glk reacting with Kct1 in different crotonyl-CoA concentration (10 mM, 15 mM and 20 mM respectively).

b. Immuno-blots analysis of Glk decrotonylation by CobB *in vitro*. Lane 1 is only Glk used in this reaction. Lane 2 and 3 are Glk with CobB in different reaction time. The reaction time for decrotonylation is: lane 1, 1.5 h; lane 2, 1 h; lane 3, 1.5 h.

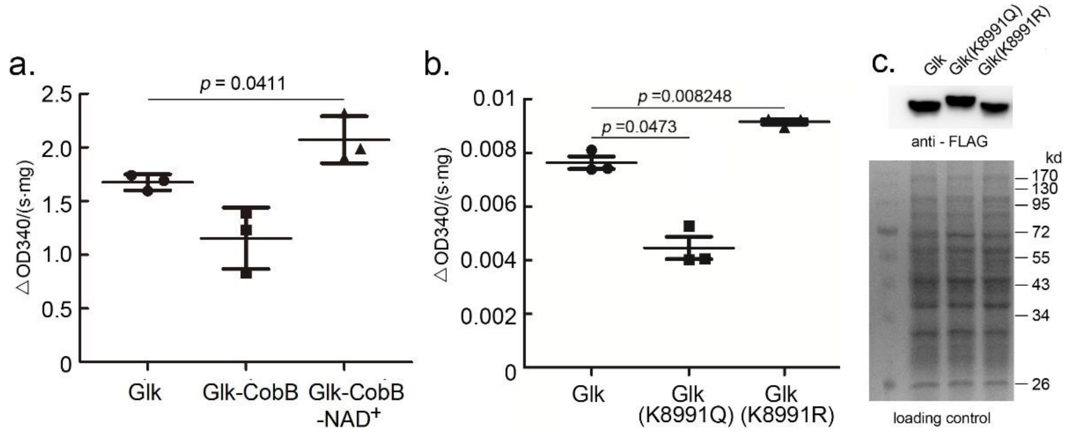

Supplementary Figure 22. Crotonylation regulates Glk activity.

a. *In vitro* glucose kinase activity assays of Glk after decrotonylation by CobB. The kinase activity assays were demonstrated with purified Glk (Glk), Glk with CobB (Glk-CobB), and Glk with both CobB and NAD^+ (Glk-CobB-NAD^+). The experiments were performed in triplicate.

b. Glucose kinase activity assays of the lysates from *Δglk* mutant complemented with Glk, Glk (K8991Q) and Glk (K8991R). Bacterial strains were cultured in the YEME medium and the kinase activity was measured after 18 h. Experiments were performed in triplicate.

c. Immuno-blot assay with anti-FLAG antibody for Glk and its mutant forms from the lysates of strains in (b). Coomassie blue staining of the total protein served as a loading control.

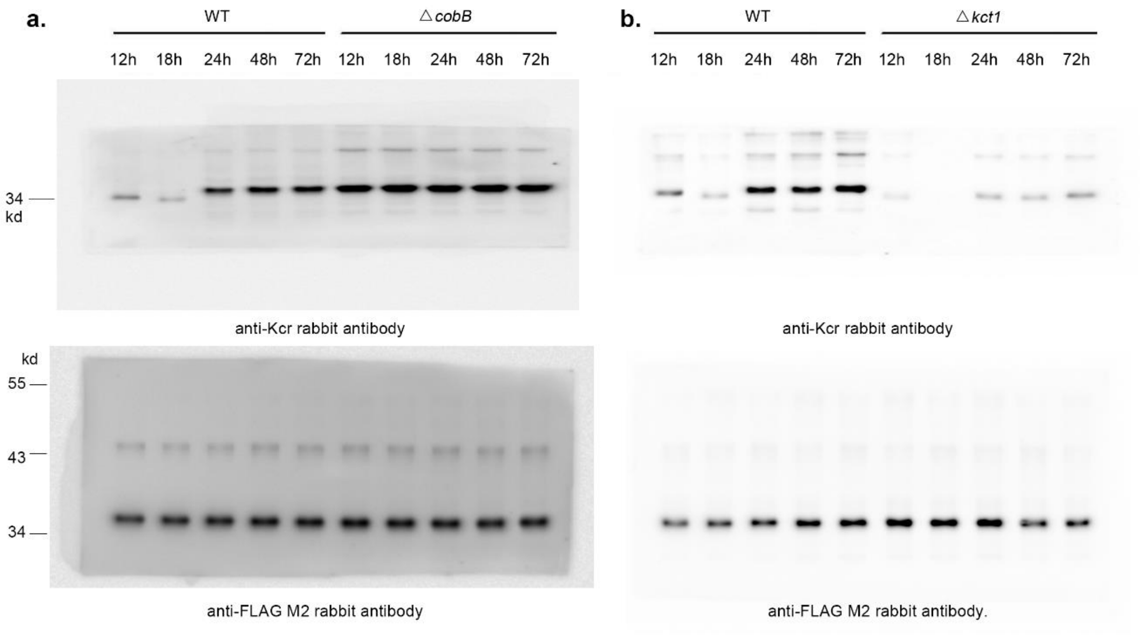

Supplementary Figure 23. Uncropped blot images of *in vivo* assays for Glk crotonylation and decrotonylation (relate to Figure 7).

Western blot analysis of crotonylation level of Glk *in vivo*. *S. roseosporus* wild type, Δ*cobB* and Δ*kct1* mutants expressing *ermEp**-3*flag-glk* were cultured in the YEME medium and mycelia were taken in the times indicated. 3FLAG-Glk was immune-precipitated from the lysate and western blots were performed with anti-FLAG M2 or anti-Kcr rabbit antibody.

Supplementary Methods

Crotonyl-proteomic analysis of *Streptomyces* proteins

Spores of *ΔprcB/A* were inoculated in TSB as the seed culture for 36 h. 1 ml of the seed transferred to 50 ml of YEME medium fermented for 3 days. All shake-flask fermentation was set at 30°C on a rotary shaker (250 rpm). The mycelia were harvested and washed twice with pre-cold PBS. The sample was grinded by liquid nitrogen into cell powder and then transferred to a 5-mL centrifuge tube. Four volumes of lysis buffer (8 M urea, 1% Triton-100, 10 mM dithiothreitol, and 1% Protease Inhibitor Cocktail) was added to the cell powder, followed by sonication for three times on ice using a high intensity ultrasonic processor (Scientz). The remaining debris was removed by centrifugation at 20,000 g at 4 ℃ for 10 min. Finally, the protein was precipitated with cold 20% TCA for 2 h at -20 ℃. After centrifugation at 12,000 g, 4 ℃ for 10 min, the supernatant was discarded. The remaining precipitate was washed with cold acetone for three times. The protein was re-dissolved in 8 M urea and the protein concentration was determined with BCA kit.

Trypsin digestion

For digestion, the protein solution was reduced with 5 mM dithiothreitol for 30 min at 56 ℃ and alkylated with 11 mM iodoacetamide for 15 min at room temperature in darkness. The protein sample was then diluted by adding 100 mM NH_4HCO_3 to urea concentration less than 2 M. Finally, trypsin was added at 1:50 trypsin-to-protein mass ratio for the first digestion overnight and 1:100 ratio for the second 4 h-digestion.

HPLC fractionation

The tryptic peptides were fractionated into fractions by high pH reverse-phase HPLC using Thermo Betasil C18 column (5 μm particles, 10 mm ID, 250 mm length). Briefly, peptides were first separated with a gradient of 8% to 32% acetonitrile (pH 9.0) over 60 min into 60 fractions. Then, the peptides were combined into 6 fractions and dried by vacuum centrifuging.

Affinity enrichment

To enrich crotonylated peptides, tryptic peptides dissolved in NETN buffer (100

mM NaCl, 1 mM EDTA, 50 mM Tris-HCl, 0.5% NP-40, pH 8.0) were incubated with pre-washed anti-Kcr antibody beads (Lot number PTM503, PTM Bio) at 4 ℃ overnight with gentle shaking. Beads were washed four times with NETN buffer and twice with H_2O. The bound peptides were eluted from beads with 0.1% trifluoroacetic acid. Finally, the eluted fractions were combined and vacuum-dried. For LC-MS/MS analysis, the resulting peptides were desalted with C18 ZipTips (Millipore).

LC-MS/MS analysis

The tryptic peptides were dissolved in 0.1% formic acid (solvent A). The gradient was comprised of an increase from 6% to 23% solvent B (0.1% formic acid in 98% acetonitrile) over 26 min, 23% to 35% in 8 min and climbing to 80% in 3 min then holding at 80% for the last 3 min, all at a constant flow rate of 400 nL/min on an EASY-nLC 1000 UPLC system.

The peptides were subjected to NSI source followed by tandem mass spectrometry (MS/MS) in Q ExactiveTM Plus (Thermo) coupled online to the UPLC. The electrospray voltage applied was 2.0 kV. The m/z scan range was 350 to 1800 for full scan, and intact peptides were detected in the Orbitrap at a resolution of 70,000. Peptides were then selected for MS/MS using NCE setting as 28 and the fragments were detected in the Orbitrap at a resolution of 17,500. A data-dependent procedure that alternated between one MS scan followed by 20 MS/MS scans with 15.0s dynamic exclusion. Automatic gain control (AGC) was set at 5E4.

Database search

The resulting MS/MS data were processed using Maxquant search engine (v.1.5.2.8). Tandem mass spectra were searched against Uniprot *Streptomyces roseosporus* database concatenated with reverse decoy database. Trypsin/P was

specified as cleavage enzyme allowing up to 4 missing cleavages. The mass tolerance for precursor ions was set as 20 ppm in First search and 5 ppm in Main search, and the mass tolerance for fragment ions was set as 0.02 Da. Carbamidomethyl on Cys was specified as fixed modification and Kcr modification and oxidation on Met were specified as variable modifications. FDR was adjusted to < 1% and minimum score for modified peptides was set > 40.
