# Supplementary material for: Crotonylation of key metabolic enzymes regulates carbon catabolite repression in Streptomyces roseosporus
Source: Commun Biol. 2020 Apr 24;3:192. doi: 10.1038/s42003-020-0924-2 (PMC7181814; doi:10.1038/s42003-020-0924-2)
Supplement: Supplementary file 2 — Description of Additional Supplementary Files [file 42003_2020_924_MOESM2_ESM.pdf]

Description of Additional Supplementary Files

File Name: **Supplementary Data 1**
Description: *All primers used in this work.*

File Name: **Supplementary Data 2**
Description: *All open reading frames of corresponding proteins in Streptomyces roseosporus.*

File Name: **Supplementary Data 3**
Description: *Analysis of crotonylated proteins and its crotonylated lysine in Streptomyces roseosporus*

File Name: **Supplementary Data 4**
Description: *Gene Ontology enrichment analysis of crotonylated proteins in Streptomyces roseosporus.*

File Name: **Supplementary Data 5**
Description: *KEGG pathway analysis for crotonylated proteins in Streptomyces roseosporus.*

File Name: **Supplementary Data 6**
Description: *Acyltransferases and deacyltransferases used for bacterial two-hybrid in this work*

File Name: **Supplementary Data 7**
Description: *All source data underlying the graphs and charts presented in the main figures.*
